# EV71 3D Protein Binds with NLRP3 and Enhances the Assembly of Inflammasome Complex

**DOI:** 10.1371/journal.ppat.1006123

**Published:** 2017-01-06

**Authors:** Wenbiao Wang, Feng Xiao, Pin Wan, Pan Pan, Yecheng Zhang, Fang Liu, Kailang Wu, Yingle Liu, Jianguo Wu

**Affiliations:** State Key Laboratory of Virology and College of Life Sciences, Wuhan University, Wuhan, China; University of Alabama at Birmingham, UNITED STATES

## Abstract

Activation of NLRP3 inflammasome is important for effective host defense against invading pathogen. Together with apoptosis-associated speck-like protein containing CARD domain (ASC), NLRP3 induces the cleavage of caspase-1 to facilitate the maturation of interleukin-1beta (IL-1β), an important pro-inflammatory cytokine. IL-1β subsequently plays critical roles in inflammatory responses by activating immune cells and inducing many secondary pro-inflammatory cytokines. Although the role of NLRP3 inflammasome in immune response is well defined, the mechanism underlying its assembly modulated by pathogen infection remains largely unknown. Here, we identified a novel mechanism by which enterovirus 71 (EV71) facilitates the assembly of NLRP3 inflammasome. Our results show that EV71 induces production and secretion of IL-1β in macrophages and peripheral blood mononuclear cells (PBMCs) through activation of NLRP3 inflammasome. EV71 replication and protein synthesis are required for NLRP3-mediated activation of IL-1β. Interestingly, EV71 3D protein, a RNA-dependent RNA polymerase (RdRp) was found to stimulate the activation of NLRP3 inflammasome, the cleavage of pro-caspase-1, and the release of IL-1β through direct binding to NLRP3. More importantly, 3D interacts with NLRP3 to facilitate the assembly of inflammasome complex by forming a 3D-NLRP3-ASC ring-like structure, resulting in the activation of IL-1β. These findings demonstrate a new role of 3D as an important player in the activation of inflammatory response, and identify a novel mechanism underlying the modulation of inflammasome assembly and function induced by pathogen invasion.

## Author Summary

The immune system protects the infected host and clears the invading pathogens. An important part of the innate immune response is the activation of NLRP3 inflammasome, which is induced upon exposure to pathogens. Activated inflammasome subsequently regulates the maturation of IL-1β that plays an important role in inflammatory response. Enterovirus 71 (EV71) is a highly contagious virus causing hand-foot-mouth disease (HFMD), meningoencephalitis, neonatal sepsis, and even fatal encephalitis in children by inducing many pro-inflammatory cytokines. Although NLRP3 inflammasome plays important role in regulating host immunity and viral infection, the assembly of NLRP3 inflammasome induced by viral infection is not known. In this study, we demonstrate that EV71 3D RNA polymerase activates NLRP3 inflammasome by binding to NLRP3. More importantly, 3D was found to interact with NLRP3 to facilitate the assembly of inflammasome complex by forming a specific ring-like structure. Therefore, these findings demonstrate a new role of viral 3D polymerase in the activation of inflammatory response, and identify a novel mechanism underlying the regulation of inflammasome assembly in responding to pathogen infection, which would provide insights into the prevention and treatment of viral infection.

## Introduction

The innate immune system is a highly conserved signaling network important for protection of the infected host and clearance of the invading pathogen [1]. Recognition of the pathogen-associate molecular patterns (PAMPS) is dependent on host pattern recognition receptors (PRRs), whose activation results in the production of interferons (IFNs) and pro-inflammatory cytokines. Several families of PRRs have been identified, including the Toll-like receptor (TLR) [2], the RIG-I-like receptor (RLR) [3], the NOD-like receptor (NLR) [4], and the C-type lectin receptor (CLR) [5].

An important part of the innate immune response is the activation of inflammasome, a cytosolic complex of proteins that activates caspase-1 (Casp-1) to produce the pro-inflammatory cytokine interleukin-1beta (IL-1β) [6]. One of the best-characterized inflammasomes consists of NLR family PYRIN domain containing-3 (NLRP3) that harbors an N-terminal PYRIN domain (PYD), a NACHT-associated domain (NAD), and a C-terminal leucine-rich repeat (LRR) [7]. The PYD domain of NLRP3 interacts with the PYD domain of the adaptor protein, apoptosis-associated speck-like protein with CARD domain (ASC). NLRP3 oligomerizes through homotypic interactions between NACHT domains following the detection of pathogen infection or cellular stress. The LRR domain has been implicated in ligand sensing and auto-regulation. NLRP3 inflammasome is activated upon exposure to pathogens, including bacteria (Listeria monocytogenes and Staphylococcus aureus) [8] and viruses (Sendai virus, adenovirus and influenza virus) [9] [10], by host-derived molecules, such as extracellular glucose [11], extracellular ATP [12], and hyaluronan [13], and also detects signs of metabolic stress and environmental irritants [7]. Together with ASC, NLRP3 promotes the cleavage of pro-Casp-1 to generate active subunits p20 and p10, which regulate the maturation of IL-1β [14]. IL-1β plays an important role in inflammatory response by recruitment and activation of immune cells as well as production of secondary pro-inflammatory cytokines [15].

NLRP3 can recognize many RNA viruses to regulate innate immunity and viral replication, including enterovirus 71 (EV71) [16]. EV71 is a highly infectious RNA virus causing hand-foot-mouth disease (HFMD), meningoencephalitis, neonatal sepsis, and even fatal encephalitis in children [17]. EV71 induces many pro-inflammatory cytokines that play important roles in the development of inflammation and associated diseases [18]. Although NLRP3 inflammasome plays important role in regulating host innate immune response and viral infection, the assembly and activation of NLRP3 inflammasome mediated by viral infection are poorly understood.

In this study, we identify a novel mechanism by which EV71 facilitates the assembly of NLRP3 inflammasome. EV71 was found to stimulate the cleavage of pro-Casp-1 and the secretion of IL-1β by modulating the components of NLRP3 inflammasome. More significantly, EV71 3D protein was shown to play a stimulatory role in the activation of NLRP3 inflammasome. 3D protein is a RNA-dependent RNA polymerase (RdRp) essential for viral replication [19]. It activates the inflammasome through direct binding to NLRP3. The interaction of 3D with NLRP3 facilitates the assembly of inflammasome complex by forming a “3D-NLRP3-ASC” ring-like structure. These findings demonstrate a novel mechanism underlying the regulation of inflammasome complex assembly in response to pathogen infection, which would provide insights into the prevention and treatment of the viral infection.

## Results

### The production and secretion of IL-1β are activated by EV71 in macrophages

**Fig 1:**
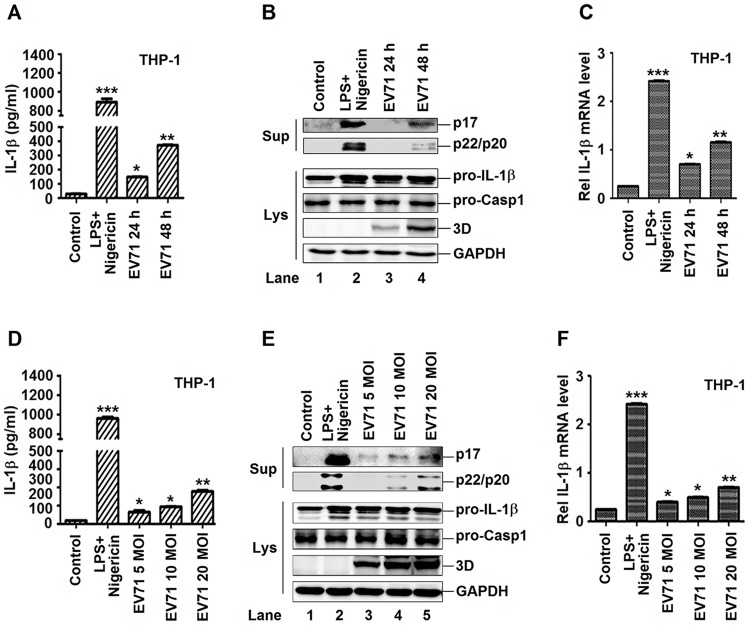
The production and secretion of IL-1β are induced by EV71 in macrophages. **(A**, **B** and **C)** TPA-differentiated THP-1 cells were infected by the EV71 virus by a time dependent (MOI = 20) for 24 h and 48 h. Supernatants were analyzed by ELISA for IL-1β secretion (A). Immunoblot analysis of the mature (p17) form of IL-1β and cleaved caspase-1 were analyzed in the supernatants (Sup). Cell lysates were normalized for protein content and analyzed by immunoblotting using antibodies specific for pro-IL-1β, pro-caspase-1, EV71 3D, and GAPDH (B). The mRNA levels for the gene pro-IL-1β were quantified by real-time PCR (C). (**D**, **E** and **F**) TPA-differentiated THP-1 cells were infected by the EV71 virus by a dose dependent (MOI = 5, 10, 20) for 24 h. Using the LPS (1 μg/ml) for 6 h and 2 μM Nigericin for 30 min as a positive control. Supernatants were analyzed by ELISA for IL-1β secretion (D). Immunoblot analysis of the mature (p17) form of IL-1β and cleaved caspase-1 in the supernatants (Sup). Cell lysates were normalized for protein content and analyzed by immunoblotting using antibodies specific for pro-IL-1β, pro-Caspase-1, and EV71 3D protein and GAPDH (Lys) (E). The mRNA levels for the gene pro-IL-1β were quantified by real-time PCR (F). Data shown are means ± SEM, *p<0.05, **p<0.01, ***p<0.0001.

EV71 infection induces pro-inflammatory cytokines that play important roles in the development of inflammation and associated diseases [20]. Among the pro-inflammatory cytokines, IL-1β plays important roles in the induction of inflammation by recruitment and activation of immune cells and production of secondary pro-inflammatory cytokines [21]. Thus, we determined the effect of EV71 on the production and secretion of IL-1β. TPA-differentiated THP-1 macrophages were infected with EV71. The secretion of IL-1β (Fig 1A) and cleavage of IL-1β (p17) and caspase-1 (p20 and p22) (Fig 1B, upper panel) in supernatants, and the production of pro-IL-1β in the cell lysates (Fig 1B, lower panel, lanes 3–4) were induced by EV71 infection. EV71 3D protein was expressed and VP1 mRNA was detected (S1A Fig) during EV71 infection (Fig 1B, lanes 3–4), indicating that EV71 replicated well in the cells. In addition, THP-1 macrophages were treated with lipopolysaccharides (LPS) and Nigericin (a bacterial toxin that activates NLRP3 by causing potassium efflux in a pannexin-1-dependent pathway) positive control. The expression of IL-1β mRNA (Fig 1C) was stimulated by LPS+Nigericin and EV71. It is clear that EV71 induces IL-β in THP-1 macrophage.

Next, TPA-differentiated THP-1 macrophages were treated with LPS and Nigericin, or infected with EV71 at different multiplicity of infections (MOI), as indicated. The secretion of IL-1β (Fig 1D) and cleavage of IL-1β (p17) and caspase-1 (p20 and p22) (Fig 1E, top panel) in the cell supernatants, and the production of pro-IL-1β in the cell lysates (Fig 1E, bottom panel, lanes 3–5) were induced by EV71 in dose-dependent manners. Similarly, the expression of IL-1β mRNA was activated by LPS/Nigericin and EV71 virus (Fig 1F). EV71 3D protein (Fig 1E) and VP1 mRNA (S1B Fig) were detected in viral infected cells, suggesting that EV71 replicated efficiently in the macrophages. Therefore, these results indicated that EV71 induces the production and secretion of IL-1β in dose-dependent manners. The activation of IL-1β is regulated by two pathways in response to pathogen infection: transcription of pre-IL-1β mRNA and proteolytical processing of pre-IL-1β protein by caspase-1 [7]. Previous study has demonstrated that EV71 infection activates NF-κB in rat vascular smooth muscle cells and SK-N-SH cells [22, 23]. We also demonstrated that the endogenous genes regulated by NF-κB were activated by EV71 and by lipopolysaccharide (LPS), an NF-κB-activating stimuli (S1C Fig). ASC oligomers were formed by EV71 infection and Nigericin treatment, indicating that EV71 and Nigericin induce inflammasome activation [24] (S1D Fig). Taken together, our results demonstrate that the production and secretion of IL-1β are induced by EV71 in macrophages.

### Activation of IL-1β by EV71 requires components of NLRP3 inflammasome

**Fig 2:**
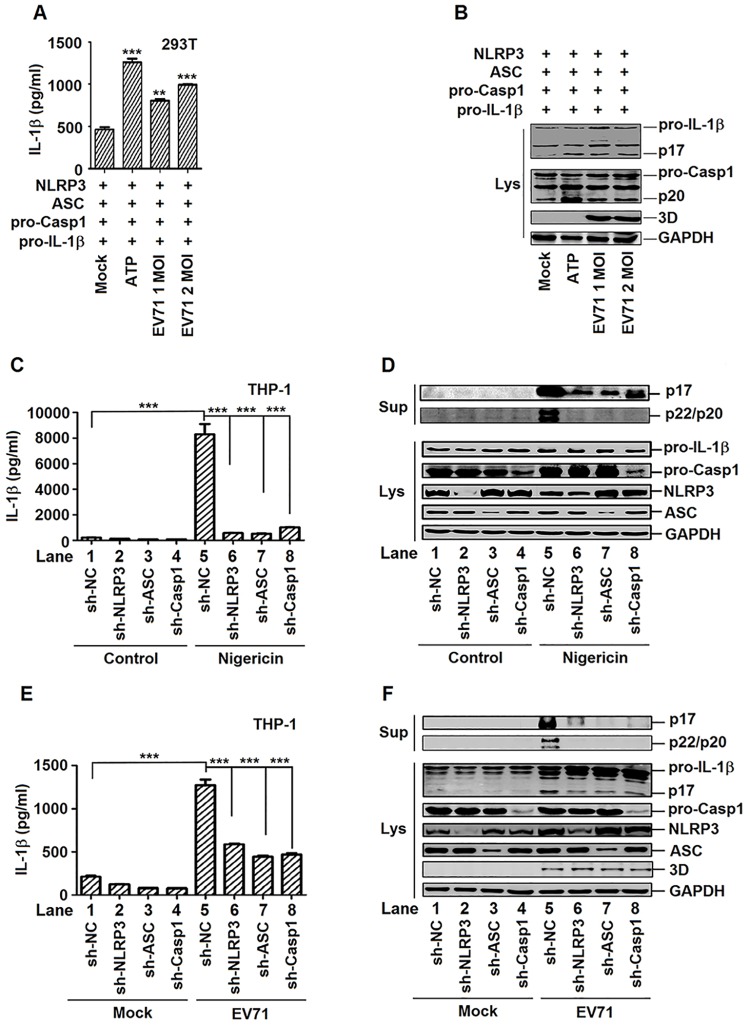
EV71 activates IL-1β through regulating the components of NLRP3 inflammasome complex. (**A** and **B**) HEK293T cells were transfected with plasmids encoding pro- IL-1β, Flag-pro-caspsase-1, Flag-NLRP3, and Flag-ASC. EV71 (MOI = 1 or 2) infected the transfected cells for 24 h. 5 mM ATP for 30 min as a positive control. Supernatants were analyzed by ELISA for IL-1β secretion (A). Cell lysates were normalized for protein content and analyzed by immunoblotting using antibodies specific to pro-IL-1β, pro-caspase-1, EV71 3D protein and GAPDH (Lys) (B). (**C** and **D**) TPA-differentiated THP-1 cells with shRNA-mediated specific gene silencing were stimulated by 2 μM Nigericin for 2 h. Supernatants were analyzed by ELISA for IL-1β secretion (C). Immunoblot analysis of the mature (p17) form of IL-1β in the supernatants (Sup). Cell lysates were normalized for protein content and analyzed by immunoblotting using antibodies specific to IL-1β, Caspase-1, NLRP3, ASC, and GAPDH (Lys) (D). (**E** and **F**) TPA-differentiated THP-1 cells with shRNA-mediated knockdown of gene expression were infected by EV71 (MOI = 20) for 24 h. Supernatants were analyzed by ELISA for IL-1β secretion (E). Immunoblot analysis of the mature (p17) form of IL-1β in the supernatants (Sup). Cell lysates were normalized for protein content and analyzed by immunoblotting using antibodies specific to IL-1β, Caspase-1, NLRP3, ASC, EV71 3D protein, and GAPDH (Lys) (F). Data shown are means±SEM, **p<0.01,***p<0.0001.

The mechanism by which EV71 activates IL-1β was investigated. As maturation of IL-1β is mediated by the NLRP3 inflammasome [6], EV71 may activate IL-1β through modulating the NLRP3 inflammasome. To determine this possibility, HEK293T cells were co-transfected with plasmids encoding the components of NLRP3 complex (NLRP3, ASC, and pro-caspsase-1) along with the substrate of NLRP3 inflammasome (pro-IL-1β), and then treated with ATP and infected with EV71. The secretion of IL-1β (p17) was up-regulated by ATP and EV71 in cell supernatants (Fig 2A), and the production of IL-1β (p17) and Casp-1 subunit (p20) were enhanced by ATP or EV71 in cell lysates (Fig 2B). These results suggest that EV71 facilitates the activation of IL-1β and Casp-1 in the presence of NLRP3 inflammasome components (Casp-1, NLRP3, and ASC) in HEK293T cells.

The roles of components of NLRP3 inflammasome complex in the production and secretion of IL-1β were further evaluated by short hairpin RNAs (shRNAs) mediated knockdown of gene expression. Four stable TPA-differentiated THP-1 macrophages cell lines were generated, which stably expressed short hairpin RNAs (shRNAs) targeting NLRP3 (sh-NLRP3), ASC (sh-ASC), and pro-caspase-1 (sh-Casp-1), respectively. In sh-NLRP3 stable cells, NLRP3 mRNA was down-regulated, whereas ASC and pro-Casp-1 mRNAs were not affected (S2A Fig). In sh-ASC stable cells, ASC mRNA was attenuated, but NLRP3 and pro-Casp-1 mRNAs remain the same (S2B Fig). In sh-Casp-1 stable cells, pro-Casp-1 mRNA was reduced, while NLRP3 and ASC mRNAs were unchanged (S2C Fig). In addition, NLRP3, ASC, and pro-Casp-1 proteins were attenuated in sh-NLRP3 stable cells, sh-ASC stable cells, and sh-Casp-1 stable cells, respectively (S2D Fig). These results indicated that shRNAs were stably expressed and specifically silenced their target gene expression. The stable cell lines were then treated with Nigericin. In the cell supernatants, secretion of IL-1β was induced by Nigericin, but its activation was significantly attenuated by sh-NLRP3, sh-ASC, and sh-Casp-1, respectively (Fig 2C). Similarly, the cleavage of IL-1β(p17) and caspase-1 (p20 and p22) were enhanced by Nigericin (Fig 2D, top panel, lane 5), but their levels were significantly down-regulated by sh-NLRP3, sh-ASC, and sh-Casp-1, respectively (Fig 2D, top panel, lanes 6–8). These results demonstrate that shRNA-mediated gene silence of the components (NLRP3, ASC, or pro-caspase-1) of NLRP3 inflammasome attenuates the production and secretion of IL-1β.

The role of NLRP3 inflammasome components in EV71-induced activation of IL-1β was further explored in the stable cell lines infected with EV71. In the cell supernatants, the secretion of IL-1β (p17) was increased by EV71, but significantly attenuated by sh-NLRP3, sh-ASC, and sh-Casp-1, respectively (Fig 2E). The cleavage of IL-1β (p17) and caspase-1 (p20 and p22) was enhanced by EV71 (Fig 2F, top panel, lane 5), but significantly reduced by sh-NLRP3, sh-ASC, or sh-Casp-1 (Fig 2F, top panel, lanes 6–8). In the cell lysates, the productions of matured IL-1β (p17) were stimulated by EV71 (Fig 2F, bottom panel, lane 5), but down-regulated by sh-NLRP3, sh-ASC, or sh-Casp-1, respectively (Fig 2F, bottom panel, lane 6–8). These results suggest that activation of IL-1β by EV71 requires the components of NLRP3 inflammasome complex. Taken together, our results demonstrate that EV71 activates IL-1β through regulating the NLRP3 inflammasome.

### EV71 replication and protein synthesis are required for the activation of IL-1β in macrophages and PBMCs

**Fig 3:**
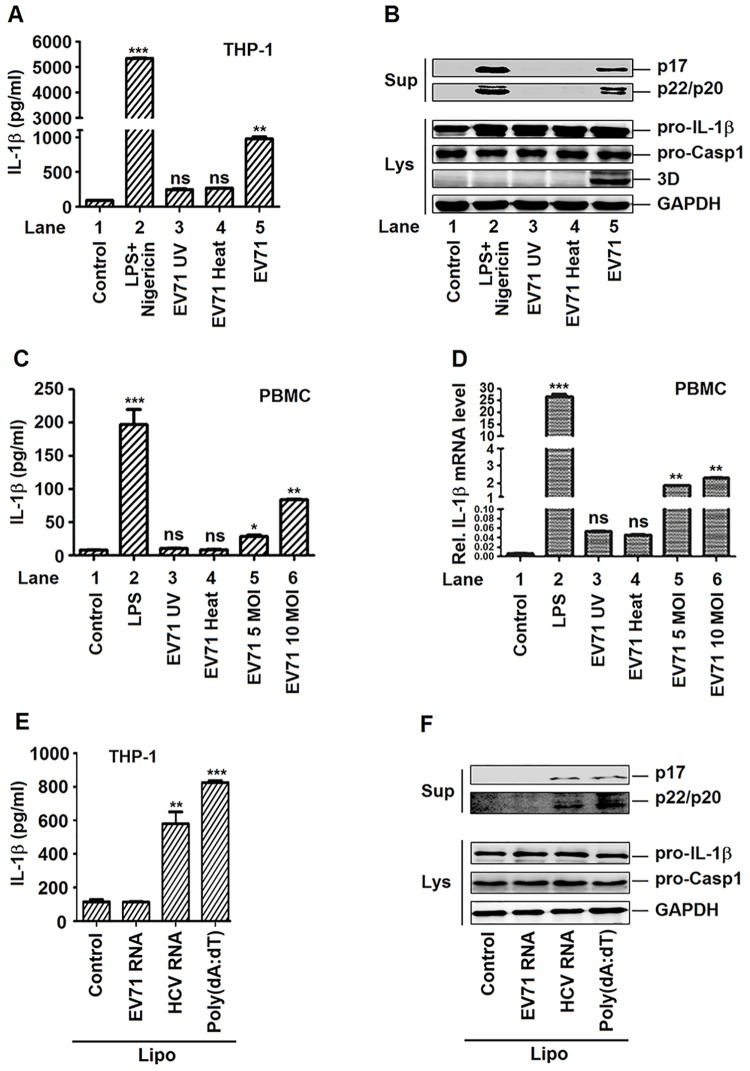
The replication of EV71 virus is required for the activation of IL-1β mediated by NLRP3. (**A** and **B**) TPA-differentiated THP-1 cells were incubated by inactivated (UV or HOT) or live EV71 virus (MOI = 20) for 48 h. The LPS (1 μg/ml) for 6 h and 2 μM Nigericin for 30 min were used as positive controls. Supernatants were used for detection of IL-1β secretion by ELISA (A). Immunoblot analysis of the mature (p17) form of IL-1β and cleaved caspase-1 in the supernatants (Sup). Cell lysates were normalized for protein content and analyzed by immunoblotting using antibodies specific to pro-IL-1β, pro-caspase-1, EV71 3D protein and GAPDH (Lys) (B). (**C** and **D**) Human PBMCs were treated with LPS (1 μg/ml) was added for 6 h, inoculated with UV- or heat-inactivated EV71 (MOI = 5) for 36 h, or infected with infectious EV71 (MOI = 5, 10) for 36 h. Supernatants were used for detection IL-1β secretion by ELISA (C). The mRNA levels of pro-IL-1β were quantified by real-time PCR (D). (**E** and **F**) TPA-differentiated THP-1 cells were stimulated for 6 h with Lipo (Control), EV71 viral RNA (5 μg/ml) plus Lipo, HCV viral RNA (5 μg/ml) plus Lipo or 5 μg/ml poly(dA:dT) plus Lipo (positive control). Supernatants were used for detection IL-1β secretion by ELISA (E). Immunoblot analysis of the mature (p17) form of IL-1β and cleaved caspase-1 in the supernatants (Sup). Cell lysates were normalized for protein content and analyzed by immunoblotting using antibodies specific to pro-IL-1β, pro-caspase-1, and GAPDH (Lys) (F). Data shown are means±SEM, *p<0.05,**p<0.01, ***p<0.0001.

The mechanism involved in the modulation of NLRP3 inflammasome mediated by EV71 was investigated. The effect of EV71 replication on the activation of IL-1β was evaluated. TPA-differentiated THP-1 macrophages were treated with LPS/Nigericin, incubated with ultraviolet (UV)-inactivated EV71 or heat-inactivated EV71, or infected with infectious EV71, as indicated. In the cell supernatants, the secretion of IL-1β was significantly increased by LPS/Nigericin and infectious EV71 and modestly up-regulated by UV- and heat-inactivated EV71 (Fig 3A). The cleavage of IL-1β (p17) and caspase-1 (p20 and p22) were significantly enhanced by LPS/Nigericin and infectious EV71, but not affected by UV- and heat-inactivated EV71 in the cell supernatants (Fig 3B, top panel). EV71 3D was expressed only in the cells infected with infectious EV71 (Fig 3B, bottom panel, lane 5), but not detected in the cells inoculated with UV- and heat-inactivated EV71 (Fig 3B, lanes 3–4), suggesting that UV- and heat-inactivated EV71 failed to replicate in the cells. Therefore, these results indicate that the replication of EV71 is required for the activation of IL-1β in macrophages.

Additionally, the effect of EV71 replication on the activation of IL-1β in human primary peripheral blood mononuclear cells (PBMCs) was evaluated. PBMCs were infected with EV71, treated with LPS, and inoculated with UV-inactivated or heat-inactivated EV71, respectively. EV71 VP1 mRNA was detected in the cells infected with EV71, but not in the cells treated with LPS, or inoculated with UV-inactivated or heat-inactivated EV71 (S3A Fig), indicating that EV71 replicates in PBMCs, but UV-inactivated EV71 and heat-inactivated EV71 failed to replicate. The secretion of IL-1β was stimulated by LPS, activated by EV71 in an MOI-dependent manner, but not affected by UV-inactivated or heat-inactivated EV71 (Fig 3C). The expression of IL-1β mRNA was stimulated by LPS, activated by EV71, but not by UV-inactivated or heat-inactivated EV71 (Fig 3D). These results suggested that the replication of EV71 is required for the production and secretion of IL-1β in PBMCs.

It is well established that NLRP3 inflammasome mediated innate immunity to virus through recognition of viral RNA [25, 26]. To further determine whether EV71 RNA alone can initiate the activation of the NLR3 inflammasome, we stimulated THP-1 macrophages with EV71 viral genomic RNA, HCV viral genomic RNA, and poly(dA:dT) as a positive control. The results showed that IL-1β secretion was induced by HCV genomic RNA and poly(dA:dT), but not by EV71 genomic RNA in the cell supernatants of THP-1 macrophages (Fig 3E). The cleavage of IL-1β (p17) and caspase-1 (p20 and p22) were not detected in the presence of transfected EV71 RNA (Fig 3F). The levels of pro-IL-1β mRNA and TNF-α mRNA were enhanced by the treatment of EV71 viral genomic RNA and HCV viral genomic RNA (S3B Fig). These results demonstrated that, unlike HCV genomic RNA and influenza A virus RNA, EV71 genomic RNA is unable to activate NLRP3 inflammasome.

### Activation of IL-1β is stimulated by the 3D protein of EV71

**Fig 4:**
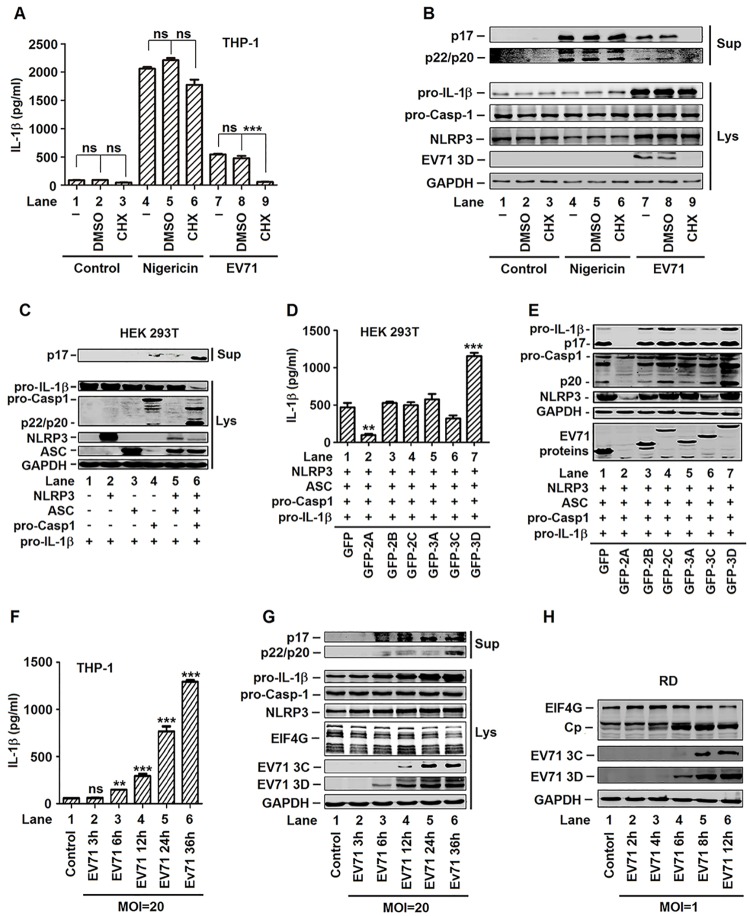
EV71 proteins was participated in the activation of NLRP3 inflammasome. (**A** and **B**) TPA-differentiated THP-1 cells were mock-infected for 2 h (lanes 1–3) or infection with EV71 (MOI = 20) for 2 h (lanes 7–9), treated with 100 μM of cycloheximide (CHX) for 1 h, washed with PBS, and then grown for additional 22 h. TPA-differentiated THP-1 cells were treated with 100 μM of cycloheximide (CHX) for 1 h, washed with PBS, grown for additional 20 h, and then treated with 2 μM Nigericin for 2 h as a positive control (lanes 4–6). Supernatants of the cell cultures were analyzed by ELISA for IL-1β secretion (A). Immunoblot analysis of the mature (p17) form of IL-1β in the supernatants (Sup). Cell lysates were normalized for protein content and analyzed by immunoblotting using antibodies specific for pro-IL-1β, pro-Casp-1, NLRP3, EV71 3D, and GAPDH proteins (Lys) (B). (**C**) HEK293T cells were transfected with plasmids encoding pro-IL-1β, with(+) or without(-) Flag-pro-caspsase-1, with(+) or without(-) Flag-NLRP3 and with(+) or without(-) Flag-ASC to established NLRP3 inflammasome. Immunoblot analysis of the mature (p17) form of IL-1β in the supernatants (Sup). Cell lysates were normalized for protein content and analyzed by immunoblotting using antibodies specific for IL-1β, Caspase-1, NLRP3, ASC, and GAPDH (Lys). (**D** and **E**) HEK293T cells were transfected with plasmids encoding pro-IL-1β, Flag-pro-Casp-1, Flag-NLRP3, Flag-ASC, and EV71 non-structure proteins (2A, 2B, 2C, 3A, 3C, and 3D). Using the GFP as a negative control. Supernatants were analyzed by ELISA for IL-1β secretion (D). Cell lysates were normalized for protein content and analyzed by immunoblotting using antibodies specific for pro-IL-1β, pro-caspase-1, NLRP3, GFP and GAPDH (E). (**F** and **G**) TPA-differentiated THP-1 cells was infected by the EV71 by a time dependent (MOI = 20) for 3, 6, 12, 24, and 36 h. Supernatants were analyzed by ELISA for IL-1β secretion (F). Immunoblot analysis of the mature (p17) form of IL-1β and cleaved caspase-1 in the supernatants (Sup). Cell lysates were normalized for protein content and analyzed by immunoblotting using antibodies specific for IL-1β, Caspase-1, NLRP3, EIF4G, EV71 3C, EC71 3D, and GAPDH proteins (Lys) (G). (**H**) RD cells was infected with EV71 at MOI = 1 for 2, 4, 6, 8, and 12 h. Cell lysates were normalized for protein content and analyzed by immunoblotting using antibodies specific for EIF4G, EV71 3C, EC71 3D, and GAPDH proteins (H). Data shown are means ± SEM, *p<0.05, **p<0.01, ***p<0.0001.

Previous study has demonstrated that rapid NLRP3 inflammasome activation is independent of “priming”, given that both NF-κB activation and new protein synthesis are not necessary [27]. CHX-pretreated cells produced IL-1β normally in response to extracellular ATP which indicated that NLRP3 inflammasome activation by ATP stimulation does not require *de novo* translation. In contrast, pretreatment of cells with cycloheximide (CHX), a protein synthesis inhibitor, significantly inhibited EMCV-induced IL-1β secretion. This indicated that virus-encoded proteins activate the NLRP3 inflammasome [28]. We further determined whether EV71 viral protein synthesis was also required for the activation of IL-1β. TPA-differentiated THP-1 macrophages were infected with EV71 for 2 h, treated with CHX for 1 h, and then grown for additional 22 h. In the absence of CHX, the secretion of IL-1β was stimulated by Nigericin treatment (Fig 4A, lane 4) or EV71 infection (Fig 4A, lane 7). In the presence of CHX, Nigericin-induced secretion of IL-1β was relatively unaffected (Fig 4A, lane 6), but EV71-activated secretion of IL-1β was significantly repressed (Fig 4A, lane 9). In the absence of CHX, the cleavages of IL-1β (p17) and caspase-1 (p20 and p22) were stimulated by Nigericin treatment (Fig 4B, lane 4) or by EV71 infection (Fig 4B, lane 7) in the cell supernatants. In the presence of CHX, Nigericin-activated cleavages of IL-1β (p17) and caspase-1 (p20 and p22) were relatively unaffected (Fig 4B, lane 6), whereas EV71-activated cleavages of IL-1β (p17) and caspase-1 (p20 and p22) were significantly reduced (Fig 4B, lane 9). The production of EV71 3D (Fig 4B, lanes 7–9) was significantly attenuated in the presence of CHX (Fig 4B, lane 9). Therefore, the activation of IL-1β mediated by Nigericin do not require de novo protein synthesis, whereas the activation of IL-1β induced by EV71 requires de novo protein synthesis. Taken together, we demonstrated that EV71 replication and protein synthesis are essential for the activation of IL-1β in macrophages and PBMCs.

The roles of EV71 nonstructural proteins in the regulation of NLRP3 inflammasome were further determined, as EV71 protein synthesis is required for such regulation. Initially, the activity of NLRP3 inflammasome was determined in HEK293T cells co-transfected together with plasmids encoding NLRP3, ASC, pro-Casp-1, and pro-IL-1β. In the cell supernatants, the secretion of IL-1β (p17) was not detected in the presence of NLRP3 alone (Fig 4C, lane 2) or ASC alone (Fig 4C, lane 3), detected at lower level in the presence of Casp-1 (Fig 4C, lane 4) or NLRP3 plus ASC (Fig 4C, lane 5), and significantly activated in the presence of all three components, NLRP3, ASC, and Casp-1 (Fig 4C, lane 6). In the cell lysates, the levels of pro-IL-1β and Casp-1 (p22/p20) were relatively unchanged in the presence of one or two components (Fig 4C, lanes 1–5), but significantly stimulated in the presence of all three components (Fig 4C, lane 6). These results indicated that all components of NLRP3 inflammasome are required for the function of the inflammasome, and suggested that NLRP3 inflammasome is functionally effective under these conditions.

HEK293T cells were co-transfected together with plasmids encoding NLRP3, ASC, pro-Casp-1, pro-IL-1β, and then transfected with each of the EV71 non-structure proteins, 2A, 2B, 2C, 3A, 3C, and 3D, respectively, as indicated. The secretion of IL-1β in the cell supernatants was inhibited by 2A, relatively unaffected by 2B and 3A, down-regulated by 2C and 3C, and activated by 3D (Fig 4D). Similarly, the production of IL-1β (p17) in the cell lysates was inhibited by 2A (Fig 4E, lane 2), unaffected by 2B and 3A (Fig 4E, lanes 3 and 5), enhanced by 2C (Fig 4E, lane 4), reduced by 3C (Fig 4E, lane 6), but activated by 3D (Fig 4E, lane 7). In addition, the cleavage of caspsase-1 (p20) was inhibited by 2A (Fig 4E, lane 2), unaffected by 2B and 2C (Fig 4E, lanes 3 and 4), reduced by 3A and 3C (Fig 4E, lanes 5 and 6), but significantly stimulated by 3D (Fig 4E, lane 7). Moreover, NLRP3 was inhibited by 2A (Fig 4E, lane 2), unaffected by 2B, 2C, and 3A (Fig 4E, lanes 3–5), repressed by 3C (Fig 4E, lane 6), and enhanced by 3D (Fig 4E, lane 7). These results revealed that EV71 2A and 3C repress the production and secretion of IL-1β through attenuating NLRP3 inflammasome, which is consistent with a previous report [16]. More interestingly, our results demonstrated that EV71 3D was the only viral protein that stimulates the secretion of IL-1β (Fig 4D and 4E) and the cleavage of pro-Casp-1 (Fig 4E). The correlation between the opposite functions of EV71 proteins was then evaluated in TPA-differentiated THP-1 cells infected with EV71 at MOI = 20 for different time. The results showed that the secretion of IL-1β was induced by EV71 at 6 h p.i. (Fig 4F, lane 3) and then increased as the infection time increased (Fig 4F, lanes 4–6). The cleavages of IL-1β (p17) and caspase-1 (p20 and p22) were stimulated by EV71 at 6 h p.i. (Fig 4G, lane 3). Similarly, EV71 3D protein was also detected at 6 h p.i. (Fig 4G, lane 3) and then increased as the infection time increased (Fig 4G, lanes 4–6). However, EV71 3C protein was detected at 12 h p.i. (Fig 4G, lane 4) and then increased as the infection time increased (Fig 4G, lanes 5 and 6). EV71 2A protein was not detect by western blot, likely due to the concomitant restriction on its expression from its inhibition effect on host gene expression [29]. Thus, EIF4G, a known substrate of 2A, was used as an indictor for the activity of 2A proteases. The results showed that EIF4G protein was reduced at 36 h p.i. (Fig 4G, lane 6). The temporal expression of EV71 2A, 3C, and 3D were further confirmed in RD cells infected with EV71 at different times. The results showed that 3D expression was initiated at 6 h p.i. (Fig 4H, lane 4), whereas EIF4G cleavage and 3C expression was started at 12 h p.i. (Fig 4H, lane 5). Taken together, these results revealed that the secretion of IL-1β or the activation of NLRP3 inflammasome was stimulated by EV71 3D and that 3D protein can overcome the inhibitory effects of EV71 2A and 3C proteins in the secretion of IL-1β. Therefore, we focused the rest of study on determining the role of EV71 3D in the activation of NLRP3 inflammasome and the mechanism underlying such regulation.

### EV71 3D stimulates the activation of NLRP3 inflammasome

**Fig 5:**
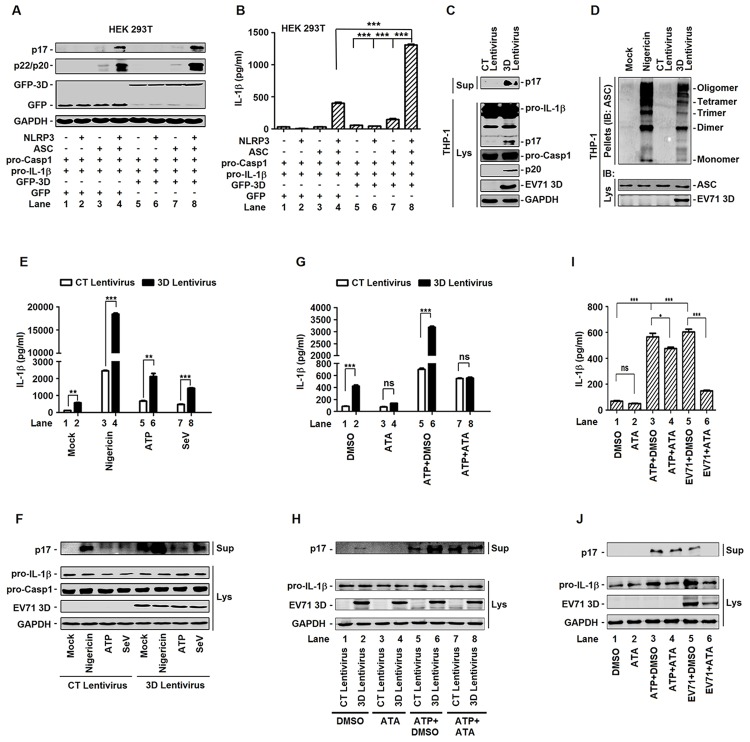
EV71 3D facilitates the activation of NLRP3 inflammasome. (**A** and **B**) HEK293T cells were transfected with plasmids encoding pro-IL-1βFlag-pro-caspsase-1, with(+) or without(-) Flag-NLRP3, with(+) or without(-) Flag-ASC and with(+) or without(-) EV71 3D protein. Using the GFP as a negative control. Cell lysates were normalized for protein content and analyzed by immunoblotting using antibodies specific for IL-1β, Caspase-1, GFP, and GAPDH (A). Supernatants of the cell cultures were analyzed by ELISA for the detection of IL-1β secretion (B). (**C**) THP-1 cells were infected by control (CT) or 3D-encoding lentivirus. And then THP-1 cells were differentiated into macrophages with TPA. The mature (p17) form of IL-1β in the supernatants were analyzed by immunoblot (Sup). Cell lysates were normalized for protein content and analyzed by immunoblotting using antibodies specific for IL-1β, Caspase-1, EV71 3D protein, and GAPDH (Lys). (**D**) ASC oligomerization in TPA-differentiated THP-1 cells which were infected by control (CT) or 3D-encoding lentivirus. TPA-differentiated THP-1 cells were treated with the 2 μM Nigericin for 2 h as a positive control. TPA-differentiated THP-1 cells were treated with DMSO as a negative control. (**E** and **F**) TPA-differentiated THP-1 cells which were infected by control (CT) or 3D-encoding lentivirus was treated with 2 μM Nigericin for 2 h, 5 mM ATP for 2 h or infection with Sendaivirus (MOI = 1) for 24 h. Supernatants were analyzed by ELISA for IL-1β secretion (E). Immunoblot analysis of the mature (p17) form of IL-1β in the supernatants (Sup). Cell lysates were normalized for protein content and analyzed by immunoblotting using antibodies specific for pro-IL-1β, pro-caspase-1, EV71 3D protein, and GAPDH (Lys) (F). (**G** and **H**) TPA-differentiated THP-1 cells were infected with control (CT) or 3D-encoding lentivirus, treated with DMSO or 80 μM aurintricarboxylic acid (ATA) for 24 h, and treated with 5 mM ATP for 2 h. Supernatants were analyzed by ELISA for IL-1β secretion (G). Immunoblot analysis of the mature (p17) form of IL-1β in the supernatants (Sup). Cell lysates were normalized for protein content and analyzed by immunoblotting using antibodies specific for pro-IL-1β, EV71 3D, and GAPDH proteins (Lys) (H). (**I** and **J**) TPA-differentiated THP-1 cells were infected with EV71 at MOI = 20 for 2 h, treated with DMSO or 80 μM aurintricarboxylic acid (ATA), and the treated with 5 mM ATP for 2 h. At 24 h post-infection of EV71, supernatants were analyzed by ELISA for IL-1β secretion (I). Immunoblot analysis of the mature (p17) form of IL-1β in the supernatants (Sup). Cell lysates were normalized for protein content and analyzed by immunoblotting using antibodies specific for pro-IL-1β, EV71 3D, and GAPDH proteins (Lys) (J). Data shown are means ± SEM, *p<0.05, **p<0.01, ***p<0.0001.

EV71 3D induces the cleavage of pro-Casp-1 and the secretion of IL-1β through suggesting that it may activate NLRP3 inflammasome, as the NLRP3 inflammasome is critical for pro-Casp-1 activation and pro-IL-1β procession. The effect of EV71 3D on the activation of NLRP3 inflammasome was determined. HEK293T cells were co-transfected together with plasmids encoding NLRP3, ASC, pro-Casp-1, and pro-IL-1β, along with plasmid encoding EV71 3D, as indicated. The productions of IL-1β (p17) and Casp-1 (p22/p20) were not detected in the presence of one or two components of the inflammasome (Fig 5A, lanes 1–3 and 5–7), activated in the presence of NLRP3, ASC, and pro-Casp-1 (Fig 5A, lane 4), and further facilitated by 3D (Fig 5A, lane 8). Similarly, the secretion of IL-1β was not detected in the presence of one or two components of the inflammasome (Fig 5B, lanes 1–3 and 5–7), stimulated in the presence of NLRP3, ASC, and pro-Casp-1 (Fig 5B, lane 4), and further enhanced by 3D (Fig 5B, lane 8). These results demonstrated that EV71 3D enhances the activity of NLRP3 inflammasome to facilitate the production and release of IL-1β.

In addition, the role of EV71 3D in regulating the activity of NLRP3 inflammasome was evaluated in TPA-differentiated THP-1 macrophages which infected with lentivirus expressing EV71 3D protein for the stable protein expression cell lines. The results showed that 3D activated the secretion of IL-1β (p17) in the cell supernatants (Fig 5C, top panel), as well as the production of IL-1β (p17) and Casp-1 (p22) in the cell lysates (Fig 5C, bottom). Furthermore, the effect of EV71 3D on ASC pyroptosome formation was evaluated in TPA-differentiated THP-1 macrophages infected with lentiviruses expressing 3D. We used the TPA-differentiated THP-1 macrophages as a negative control and the TPA-differentiated THP-1 macrophages which were treated with Nigericin as a positive control. ASC pyroptosome formation was activated by 3D and Nigericin, respectively (Fig 5D). In addition, the role of EV71 in regulating the activity of NLRP3 inflammasome was evaluated in TPA-differentiated THP-1 macrophages infected with lentiviruses expressing EV71 3D, treated with Nigericin or ATP, and infected with Sendai virus (SeV). The secretion of IL-1β (p17) was activated by Nigericin, ATP, and SeV in the cell supernatants, whereas such activations were significantly facilitated by 3D (Fig 5E). Similarly, the secretion of IL-1β (p17) was stimulated by Nigericin, ATP and SeV, whereas 3D further facilitated the secretion of IL-1β (p17) mediated by Nigericin, ATP, and SeV (Fig 5F).

Previous study has demonstrated that aurintricarboxylic acid (ATA) was able to inhibit the RdRp activity of EV71 3D protein, but not inhibit the protease activities of 2A and 3C [30]. Our results revealed that lentivirus expressed EV71 3D activated the secretion of IL-1β in THP-1 cells in the absence of ATA (Fig 5G, lane 2), but failed to induce the secretion of IL-1β in the presence of ATA (Fig 5G, lane 4). In the absence of ATA, the secretion of IL-1β (p17) was activated by ATP (Fig 5G, lane 5) and such activation was significantly facilitated by 3D (Fig 5G, lane 6). In the presence of ATA, the secretion of IL-1β (p17) was also activated by ATP (Fig 5G, lane 7), but EV71 3D had no affect on ATP-induced secretion of IL-1β (Fig 5G, lane 8). Similarly, EV71 3D protein stimulated the cleavage of IL-1β (p17) in the absence of ATA (Fig 5H, lane 2), but failed to induced the cleavage of IL-1β (p17) in the presence of ATA (Fig 5H, lane 4). In the presence of ATA, the cleavage of IL-1β was induced by ATP (Fig 5H, lane 7), but EV71 3D failed to facilitate ATP-induced secretion of IL-1β (Fig 5H, lane 8). In addition, 3D protein production was not affected by ATA (Fig 5H, lane 2 *vs* 4, and lane 6 *vs* 8), suggesting that the RdRp activity of EV71 3D may be required for the activation of NLRP3 inflammasome. Moreover, TPA-differentiated THP-1 macrophages were infected with EV71 and treated with ATA and ATP. The results showed that ATP-induced secretion of IL-1β (Fig 5I, lane 3) was slightly reduced by ATA (Fig 5I, lane 3), but EV71-induced secretion of IL-1β (Fig 5I, lane 5) were significant attenuated by ATA (Fig 5I, lane 6). Similarly, ATP-induced cleavage of IL-1β (Fig 5J, lane 3) was slightly reduced by ATA (Fig 5J, lane 4), but EV71-induced cleavage of IL-1β (Fig 5J, lane 5) were significant attenuated by ATA (Fig 5J, lane 6). These results demonstrated that RdRp activity of EV71 3D is essential for NLRP3 inflammasome activation. Taken together, we revealed that EV71 3D protein induces the activity of NLRP3 inflammasome and the productions of IL-1β (p17) and Casp-1 (p22/p20), and that the RdRp activity of EV71 3D is essential for EV71-induced activation of NLRP3 inflammasome.

### NLRP3 binds with EV71 3D through NACHT and LRR domains

**Fig 6:**
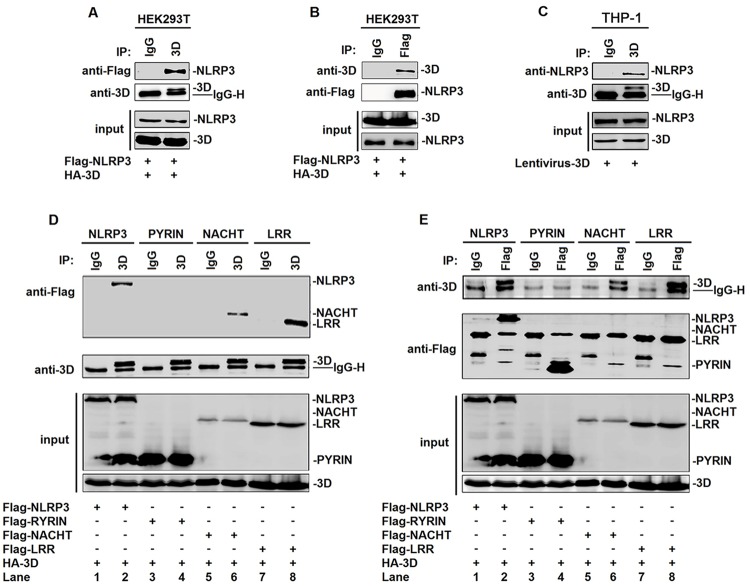
NLRP3 interacts with EV71 3D through the NACHT and LRR domains. (**A** and **B**) Human embryonic kidney cells (HEK 293T) were transfected with HA-3D plasmid in combination with plasmids encoding Flag-NLRP3. Lysates were either analyzed directly (30 μg protein; bottom) by immunoblotting using anti-3D and anti-Flag antibody (input) or subjected to IP using control IgG or anti-3D antibody (A) or control IgG and anti-Flag antibody (B) and then analyzed by immunoblotting using anti-3D and anti-Flag antibody. (**C**) THP-1 cells were infected by control (CT) or 3D-encoding lentivirus. And then THP-1 cells were differentiated into macrophages with TPA. Lysates were either analyzed directly (30 μg protein; bottom) by immunoblotting using anti-NLRP3 and anti-3D antibody (input) or subjected to IP using control IgG or anti-3D (top) antibody and then analyzed by immunoblotting using anti-NLRP3 and anti-3D antibody. (**D** and **E**) HEK293T cells were transfected with HA-3D plasmid in combination with plasmids encoding Flag-NLRP3, Flag-PYRIN, Flag-NACHT, or Flag-LRR. Lysates were either analyzed directly (30 μg protein; bottom) by immunoblotting using anti-3D and anti-Flag antibody (input) or subjected to IP using control IgG and anti-3D antibody (D) or control IgG and anti-Flag antibody (E) and then analyzed by immunoblotting using anti-3D and anti-Flag antibody.

The mechanism underlying the regulation of NLRP3 inflammasome mediated by EV71 3D was elucidated. Initially, we determined whether EV71 3D is interacted with the components of NLRP3 inflammasome. Interestingly, yeast strain AH109 was co-transformed with the combination of AD-3D and BD-NLRP3 inflammasome components or the three functional domains of NLRP3. We revealed that EV71 3D was interacted with NLRP3 LRR domain (S4A and S4B Fig). The interaction between EV71 3D and NLRP3 was verified by co-immunoprecipitation (CoIP) assays in HEK293T cells co-transfected with plasmid expressing Flag-NLRP3 or HA-3D, as indicated. CoIP results showed that 3D was associated with NLRP3 (Fig 6A) and NLRP3 was interacted with 3D (Fig 6B). The interaction between EV71 3D and NLRP3 was further determined in TPA-differentiated THP-1 macrophages infected with lentiviruses expressing 3D. CoIP results indicated that 3D was also interacted with NLRP3 in the treated macrophages (Fig 6C). These results demonstrated that EV71 3D protein can interact with NLRP3 protein.

NLRP3 contains several prototypic domains, including PYD, NACHT, and LRR domains. Four plasmids expressing NLRP3, PYRIN domain, NACHT domain, and LRR domain were constructed (S4C Fig). The domains of NLRP3 involved in the interaction with EV71 3D were then determined. HEK293T cells were co-transfected with plasmid expressing HA-3D along with plasmids expressing Flag-NLRP3, Flag-PYD, Flag-NACHT, and Flag-LRR, respectively. CoIP results showed that 3D was interacted with NLRP3 (Fig 6D, lane 2), NACHT domain (Fig 6D, lane 6), and LRR domain (Fig 6D, lane 8), but not PYD domain (Fig 6D, lane 4). Similarly, NLRP3 (Fig 6E, lane 2), NACHT domain (Fig 6E, lane 6), and LRR domain (Fig 6E, lane 8), but not PYD domain (Fig 6E, lane 4), were interacted with 3D. Therefore, we demonstrated that EV71 3D binds with NLRP3 through interacting with the NACHT and LRR domains.

### EV71 3D is associated with the components of inflammasome complex through interacting with NLRP3

**Fig 7:**
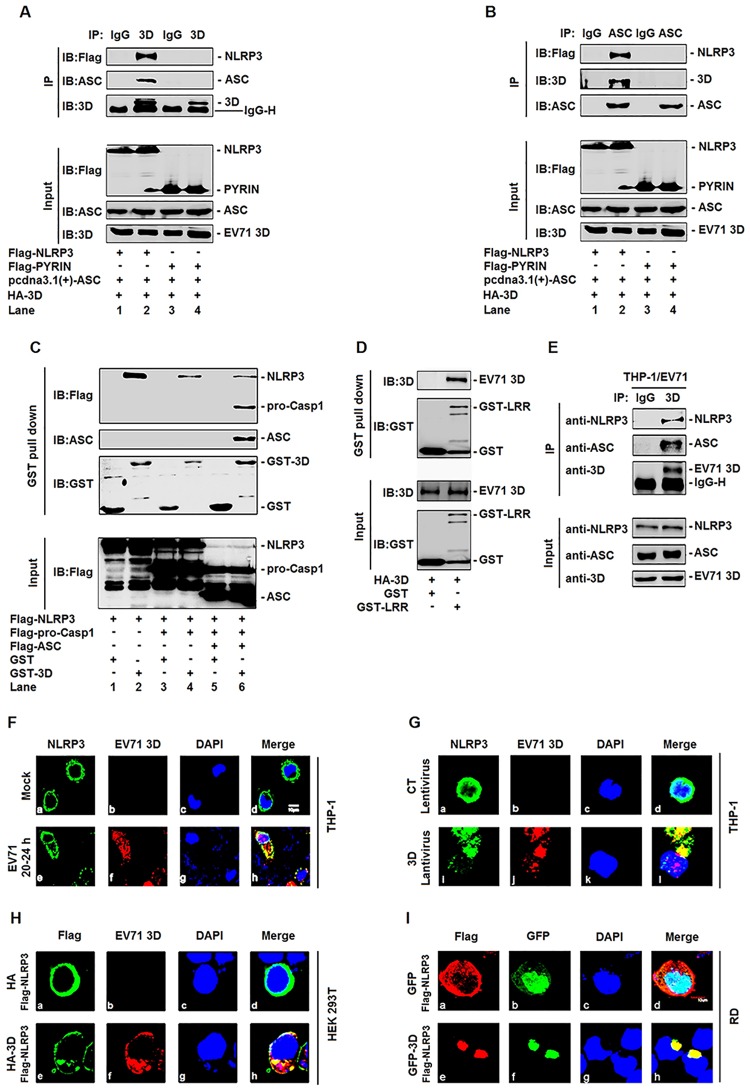
EV71 3D associates with inflammasome complex through interacting with NLRP3. (**A** and **B**) HEK293T cells were transfected with HA-3D and ASC plasmid in combination with plasmids encoding Flag-NLRP3 or Flag-PYRIN. Lysates were either analyzed directly (30 μg protein, bottom) by immunoblotting using anti-Flag, anti-ASC, and anti-3D antibody (input) or subjected to IP using control IgG and anti-3D antibody (A) or control IgG and anti-ASC antibody (B) and then analyzed by immunoblotting using anti-Flag, anti-ASC and anti-3D antibody (top). (**C**) Extracts from HEK293T cells transfected with Flag-NLRP3, with(+) or without(-) Flag-pro-caspase-1, with(+) or without(-) Flag-ASC were incubated with 10 μg GST protein or GST-3D protein which was incubated with glutathione-Sepharose beads. Mixture were washed three times and then analyzed by immunoblotting using anti-Flag, anti-ASC and anti-GST antibody (top). Lysates from transfected HEK293T cells were analyzed by immunoblotting using anti-Flag antibody. (**D**) Extracts from HEK293T cells transfected with HA-3D were incubated with 10 μg GST protein or GST-LRR protein which was incubated with glutathione-Sepharose beads. Mixture were washed three times and then analyzed by immunoblotting using anti-3D and anti-GST antibody (top). Lysates from transfected HEK293T cells and the purified proteins were analyzed by immunoblotting using anti-Flag and anti-GST antibody (bottom). (**E**) TPA-differentiated THP-1 cells was infected by the EV71 (MOI = 20) for 24 h. Lysates were either analyzed directly (30 μg protein, bottom) by immunoblotting using anti-NLRP3, anti-ASC, and anti-3D antibody (input) or subjected to IP using control IgG or anti-3D (top) antibody and then analyzed by immunoblotting using anti-NLRP3, anti-ASC and anti-3D antibody. (**F**) TPA-differentiated THP-1 macrophages were infected with EV71 at MOI = 20 for 24 h. The sub-cellular localizations of NLRP3 (green), and 3D (red), and nucleus marker DAPI (blue) were analyzed under confocal microscopy. (**G**) TPA-differentiated THP-1 macrophages were infected with control lentivirus or with 3D-encoding lentivirus. The sub-cellular localizations of NLRP3 (green), 3D (red), and DAPI (blue) were analyzed with confocal microscopy. (**H**) HEK293T cells were co-transfected with plasmid expressing Flag-NLRP3 and plasmid encoding HA-3D. The sub-cellular localizations of Flag-NLRP3 (green), HA-3D (red), and nucleus marker DAPI (blue) were analyzed with confocal microscopy. (**I**) RD cells were co-transfected with plasmid expressing Flag-NLRP3 and plasmid encoding GFP-3D. The sub-cellular localizations of Flag-NLRP3 (red), and HA-3D (green), and nucleus marker DAPI (blue) were analyzed with confocal microscopy.

We then determined whether 3D also interacts with other components (ASC and Casp-1) of NLRP3 inflammasome through interacting with NLRP3. Upon activation, NLRP3 is oligomerized, which leads to NLRP3 PYD domain clustering and presentation for interaction with ASC PYD domain, and ASC CARD domain subsequently recruits pro-Casp-1 CARD domain to permit the auto-cleavage and the formation of active Casp-1 p10/p20. Thus, we evaluated the ability of 3D in the interaction with ASC. CoIP assays showed that 3D was interacted with NLRP3 (S5 Fig, lane 2), but not ASC (S5 Fig, lane 4). The interaction between 3D and ASC was further investigated in HEK293T cells co-transfected with plasmids expressing NLRP3, PYRIN domain, ASC, and 3D, as indicated. 3D was associated with ASC only in the presence of NLRP3 (Fig 7A, lane 2), but not in the presence of PYRIN domain (Fig 7A, lane 4). Similarly, ASC was associated with 3D only in the presence of NLRP3 (Fig 7B, lane 2), but not in the presence of PYRIN domain (Fig 7B, lane 4). NLRP3 PYRIN domain interacts with ASC, but not with 3D, suggesting that 3D is associated with ASC through interacting with NLRP3. In addition, the interaction of 3D with NLRP3, ASC, and pro-Casp-1 was evaluated by GST pull-down assays. HEK293T cells were co-transfected with plasmid expressing Flag-NLRP3 and plasmids encoding Flag-pro-Casp-1, Flag-ASC, and GST-3D, as indicated. The results showed that 3D could interact with NLRP3 even in the absence of ASC and pro-Casp-1 (Fig 7C, lane 2), and could also interact with NLRP3 but not with pro-Casp-1 in the absence of ASC (Fig 7C, lane 4). However, 3D was associated with NLRP3, ASC, and pro-Casp-1 in the presence of all components of NLRP3 inflammasome (Fig 7C, lane 6). The purified GST-LRR could interact with HA-3D protein which also demonstrated the interaction between NLRP3 and EV71 3D protein (Fig 7D). These results indicated that 3D is associated with ASC or pro-Casp-1 through interacting with NLRP3. Moreover, the interactions of 3D with endogenous NLRP3 and ASC were explored in TPA-differentiated THP-1 macrophages infected with EV71. The results showed that 3D was co-immunoprecipitated with endogenous NLRP3 and ASC (Fig 7E). Thus, EV71 3D is associated with the components (NLRP3, ASC, and pro-Casp-1) of NLRP3 inflammasome through interacting with NLRP3.

As 3D interacts with NLRP3, the effect of 3D on the sub-cellular distribution of NLRP3 was examined under confocal microscopy. We evaluated the effect of EV71 on the sub-cellular distribution of NLRP3 in TPA-differentiated THP-1 macrophages infected with EV71 by immunofluorescence assays. In mock-infected macrophages (Fig 7Fa to 7Fd), NLRP3 was diffusely distributed in the cell cytosol (Fig 7Fa and 7Fd). However, in EV71-infected cells (Fig 7Fe to 7Fh), NLRP3 was co-localized with 3D (Fig 7Fe and 7Ff) to form distinct spots in the cell cytosol (Fig 7Fh). These results revealed that 3D interacts with NLRP3 to form a 3D-NLRP3 complex in the cytoplasm during EV71 infection. The role of EV71 on the sub-cellular distribution of ASC was also determined in TPA-differentiated THP-1 macrophages infected with or without EV71. In mock-infected macrophages (S6a to S6c Fig), ASC was diffusely distributed in the nucleus and cytosol (S6a and S6c Fig). In EV71-infected cells (S6d to S6f Fig), ASC was distributed as distinct small spots in the cytosol (S6d and S6f Fig). These results indicated that EV71 alters the sub-cellular distribution of ASC.

The effect of 3D on the sub-cellular distribution of NLRP3 was then determined in TPA-differentiated THP-1 macrophages infected with control lentivirus or 3D-encoding lentivirus. In the absence of 3D (Fig 7Ga to 7Gd), NLRP3 was diffusely distributed in the cytoplasm (Fig 7Ga and 7Gd). In the presence of 3D (Fig 7Ge to 7Gh), NLRP3 was co-localized with 3D (Fig 7Ge and 7Gf) to form large spots in the cytoplasm (Fig 7Gh). The association of 3D with the components of NLRP3 inflammasome was further examined in HEK293T cells co-transfected with plasmid expressing Flag-NLRP3 and plasmids expressing HA-3D, as indicated. In the absence of 3D, NLRP3 was diffusely distributed in the cytosol of HEK293T cells (Fig 7Ha and 7Hd); whereas in the presence of 3D, NLRP3 was co-localized with 3D (Fig 7He and 7Hf) to form large spots in the cytosol (Fig 7He). The association of 3D with the components of NLRP3 inflammasome was also determined in RD cells co-transfected with plasmid expressing Flag-NLRP3 and plasmids expressing GFP-3D, as indicated. Similarly, in the absence of 3D, NLRP3 was diffusely distributed in the cytosol of RD cells (Fig 7Ia and 7Id); but in the presence of 3D, NLRP3 was co-localized with 3D (Fig 7Ie and 7If) to form large spots in the cytosol (Fig 7Ie). These results revealed that 3D regulates the sub-cellular distribution of NLRP3 through interacting with NLRP3 in TPA-differentiated THP-1 macrophages, HEK293T cells, and RD cells. Taken together, we demonstrated that EV71 3D protein is associated with the major components (NLRP3, ASC, and pro-Casp-1) of NLRP3 inflammasome through interacting with NLRP3, and also revealed that 3D regulates the sub-cellular distributions of NLRP3 inflammasome by binding to NLRP3 and forming a 3D-NLRP3 complex. Thus, these results suggested that 3D may regulate the assembly of NLRP3 inflammasome.

### EV71 3D facilitates the assembly of inflammasome by binding to NLRP3

**Fig 8:**
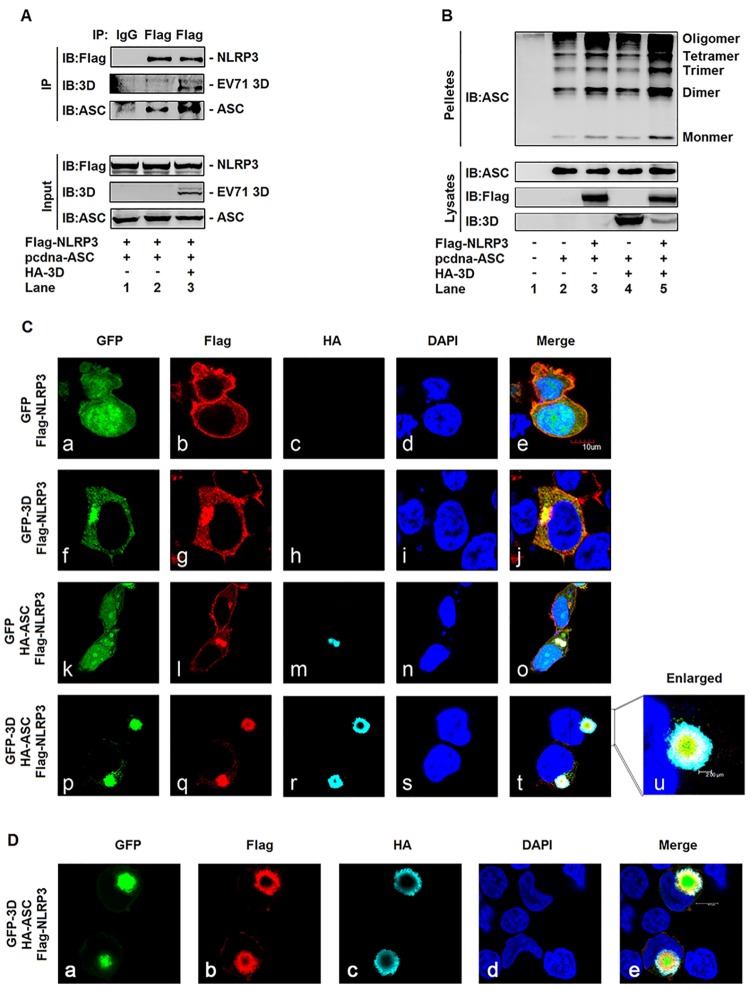
EV71 3D facilitates the assembly of inflammasome by binding to NLRP3. (**A**) HEK293T cells were transfected with the Flag-NLRP3 and pcdna3.1(+)-ASC in combination without(-) or with (+) HA-3D. Lysates were either analyzed directly (30 μg protein, bottom) by immunoblotting using anti-Flag, anti-ASC, and anti-3D antibody (input) or subjected to IP using control IgG and anti-Flag antibody, and then analyzed by immunoblotting using anti-Flag, anti-ASC and anti-3D antibody (top). (**B**) HEK293T cells were transfected with pcdna3.1(+)-ASC, Flag-NLRP3, and HA-3D for 24 h, and then harvested and performed for ASC oligomerization assay. (**C** and **D**) HEK293T cells were transfected with Flag-NLRP3 plasmid in combination with plasmids encoding with GFP, GFP-3D, or HA-ASC. The localization of nucleus marker DAPI (blue), GFP or GFP3D (green), Flag-NLRP3 (red), HA-ASC (cyan), and merge were analyzed with confocal microscopy.

In the absence of stimulation, NLRP3 is homo-oligomerized to form inactive preassembled complexes, which undergo conformational changes to form active inflammasome complexes containing ASC upon stimulation [31]. Since 3D protein is associated with the components of inflammasome through interacting with NLRP3, we speculated that 3D may facilitate the assembly of inflammasome by direct binding to NLRP3 after EV71 infection. To verify this hypothesis, HEK293T cells were co-transfected with plasmids expressing Flag-NLRP3, pcdna3.1(+)-ASC, and HA-3D, as indicated. ASC was recruited by NLRP3 (Fig 8A, lane 2), and NLRP3-mediated recruitment of ASC was significantly enhanced by 3D (Fig 8A, lane 3). The oligomerization of ASC is critical for Casp-1 activation and inflammasome function [24]. Therefore, we further investigated the effect of 3D on the oligomerization of ASC. HEK293T cells were co-transfected with plasmids expressing pcdna3.1(+)-ASC, Flag-NLRP3, and HA-3D, as indicated. The results revealed that oligomerization of ASC was detected in the presence of ASC (Fig 8B, lane 2), enhanced by NLRP3 (Fig 8B, lane 3), and not affected by 3D in the absence of NLRP3 (Fig 8B, lane 4). However, in the presence of NLRP3, oligomerization of ASC was significantly stimulated by 3D (Fig 8B, lane 5). Therefore, these results suggested that 3D facilitates the assembly of NLRP3 inflammasome through interacting with NLRP3.

It has been demonstrated that the localization of NLRP3 as spots is a character of the formation of inflammasome complex [6]. Thus, the effect of EV71 3D on the formation of NLRP3 inflammasome complex was explored in HEK293T cells co-transfected with plasmids encoding GFP, GFP-3D, Flag-NLRP3, and/or HA-ASC, as indicated. In the absence of 3D and ASC (Fig 8Ca to 8Ce), NLRP3 was diffusely distributed in the cell cytosol (Fig 8Cb and 8Ce). In the presence of 3D and absence of ASC (Fig 8Cf to 8Cj), NLRP3 was co-localized with 3D (Fig 8Cf and 8Cg) and distributed as large spots in the cell cytosol (Fig 8Cj). In the absence of 3D and presence of ASC (Fig 8Ck to 8Co), NLRP3 was co-localized with ASC (Fig 8Ck and 8Ci) and distributed as large spots in the cell cytosol (Fig 8Co). Interestingly, in the presence of both 3D and ASC (Fig 8Cp to 8Ct), NLRP3 was co-localized with 3D (Fig 8Cp and 8Cq) and ASC (Fig 8Cq and 8Cr) and distributed as large spots in the cytosol of transfected cells (Fig 8Ct). These results clearly showed that 3D, NLRP3, and ASC together formed a ring-like structure in the cytosol (Fig 8Cp, 8Cq, 8Cr and 8Ct). More interestingly, we observed that in the 3D-NLRP3-ASC complex, 3D (as indicated in green) was located inside in the ring-like structure, followed by the 3D-NLRP3 complex (green + red, as indicated in yellow) and the NLRP3-ASC complex (red + cyan, as indicated in white), and finally ASC (as indicated in cyan) was located outside the ring-like structure (Fig 8Cu, enlarged). Moreover, the formation of 3D-NLRP3-ASC complex was also observed in HEK293T cells co-transfected with plasmids expressing GFP-3D, Flag-NLRP3, and HA-ASC. The results confirmed that 3D, NLRP3, and ASC proteins together were indeed distributed as large spots in the cytosol and formed a ring-like structure (Fig 8De). In this ring-like structure, 3D was located inside (Fig 8Da), followed by NLRP3 in the middle (Fig 8Db), and ASC was located outside (Fig 8Dc). These results suggested that 3D facilitates the assembly of NLRP3 inflammasome, since it has been demonstrated that the inflammasome complex is assembled by the formation of a ring-like organization [32]. Taken together, we demonstrated that 3D facilitates the assembly of NLRP3 inflammasome complex by the formation of a “3D-NLRP3-ASC” structure through direct binding to NLRP3.

## Discussion

**Fig 9:**
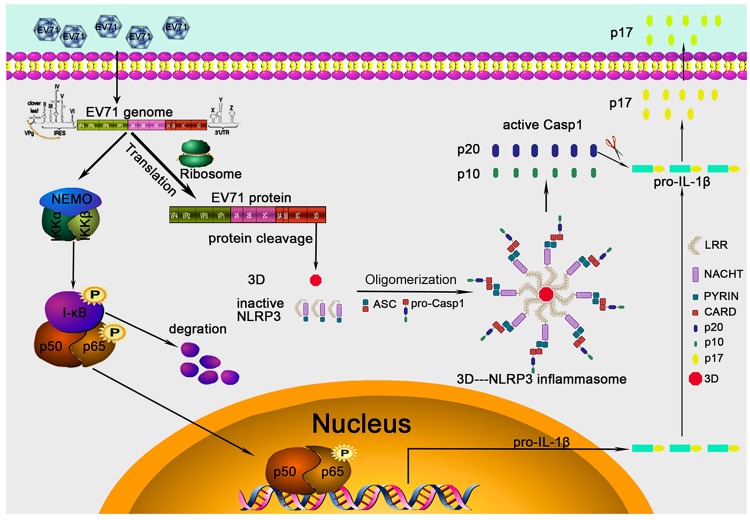
A proposed mechanism underlying EV71 3D facilitates the assembly of inflammasome by binding to NLRP3 to form a ring-like structure. During EV71 infection, the viral 3D RNA polymerase interacts directly with NLRP3 to facilitate the assembly of NLRP3 inflammasome complex by forming a “3D-NLRP3-ASC” ring-like structure. During the formation of the special structure, 3D binds to the LRR domain of NLRP3 that subsequently interacts with ASC through the PYRIN domain, ASC in turn binds to pro-Casp-1 by the CARD domain and activates Casp-1 (p20/p10), which finally stimulates the cleavage and release of IL-1β(p17). Therefore, in this specific ring-like structure, the viral protein sites in the center, followed by NLRP3 in the middle and ASC then locates outside.

The assembly of inflammasomes is critical for the host innate immune response against pathogen infection [6]. The best-characterized inflammasomes is the NLRP3 inflammasome, which activates the maturation of IL-1β through promoting the cleavage of pro-Casp-1 to generate active subunits, p20 and p10 [14]. NLRP3 inflammasome regulates innate immunity in responding to several RNA viruses, including influenza A virus (IAV) [33], encephalomyocarditis virus (EMCV) and vesicular stomatitis virus (VSV) [34], measles virus (MV) [35], myxoma virus (MYXV) [36], adenovirus (Ad) [37], West Nile virus (WNV) [38], Rabies virus (RV) [39], Herpes simplex virus 1 (HSV-1) [40], Rift valley fever virus (RVFV) [41], human T-lymphotropic virus 1 (HTLV-1) [42], and EV71 [16]. However, the mechanisms underlying the assembly of NLRP3 inflammasome regulated by viruses have not been reported. In this study, we revealed a novel mechanism by which EV71 facilitates the assembly of NLRP3 inflammasome (Fig 9).

We showed that EV71 stimulates the production of Casp-1 and the release of IL-1β in macrophages and PBMCs. EV71 induces IL-1β secretion only in the presence of all components (NLRP3, pro-Casp-1, and ASC) of NLRP3 inflammasome, and knock-down of the components attenuates EV71-induced activation of IL-1βwhich demonstrated that EV71 activates IL-1β through regulating NLRP3 inflammasome complex. The activation of NLRP3 inflammasome is regulated by two pathways in response to pathogen infection [7]. The first pathway leads to the activation of nuclear factor-kappa B (NF-κB) that subsequently induces the production of pro-IL-1β, NLRP3, and ASC. The second pathway is initiated by the assembly of inflammasome, resulting in the activation of Casp-1 and the maturation of IL-1β. IL-1β acts as an important mediator of inflammation to stimulate the recruitment and activation of immune cells and the production of many secondary pro-inflammatory cytokines [15].

We further demonstrated that UV-inactivated EV71 and heat-inactivated EV71 fail to activate IL-1β and Casp-1, suggesting that the replication of EV71 is necessary for the activation of IL-1β. Unlike HCV genomic RNA and influenza A virus RNA, EV71 genomic RNA is unable to activate NLRP3 inflammasome. In addition, the protein synthesis inhibitor CHX blocks EV71-mediated release of IL-1βindicating that viral protein synthesis is also required for the activation of IL-1β. Previous study had demonstrated that several viral proteins inhibit NLRP3 inflammsome-mediated IL-1β secretion, including MV V protein [35], IAV NS1 and M2 proteins [43, 44], and encephalomyocarditis virus (EMCV) 2B protein [28]. Our detailed studies revealed that EV71 nonstructural proteins 3D are involved in the regulation of NLRP3 inflammasome. We demonstrated that EV71 3D plays a stimulatory role in the regulation of NLRP3 inflammasome. The 3D protein acts as a viral RdRp and plays an essential role in viral negative-strand synthesis and VPg uridylylation [45]. The VPg and uridylylated forms of VPg (VPg-pUpU) prime the initiation of RNA replication [46]. Many investigators have tried to inhibit the activity of 3D protein and thereby inhibit viral replication [30, 47, 48]. It is also involved in the regulation of cell S-phase arrest [49] and immune response [50]. However, the role of 3D in the regulation of NLRP3 inflammasome has not been reported. Thus, 3D function in the activation of NLRP3 inflammasome and the mechanism underlying such regulation were intensively investigated in this study.

Interestingly, we showed that EV71 2A and 3C repress the production and secretion of IL-1β through attenuating NLRP3 inflammasome, which is consistent with a previous report [16]. More interestingly, we demonstrated that EV71 3D is the only viral protein that stimulates the secretion of IL-1β. We further revealed that 3D can directly bind with NLRP3. We revealed that 3D interacts with NLRP3 LRR domain based on the result of yeast two-hybrid analysis, and further verified the interaction of 3D with NLRP3 by several approaches, including CoIP, GST pull down, and immunofluorescence assays. NLRP3 contains three important domains, PYD, NACHT, and LRR. We confirmed that 3D can interact with NACHT and LRR domains of NLRP3. Upon activation, NLRP3 is oligomerized that leads to the interaction of NLRP3 with ASC PYRIN domain, which subsequently recruits pro-Casp-1 through CARD domain to permit the auto-cleavage and formation of active Casp-1 (p10/p20) [7]. Although 3D was not interacted directly with ASC and pro-Casp-1, it is associated with NLRP3, ASC, and pro-Casp-1, through interacting with NLRP3. To our knowledge, there was no report describing a direct interaction between 3D and NLRP3. Thus, we at the first time demonstrated that a viral RdRp interacts directly with NLRP3 to activate the inflammasome. Moreover, 3D alters the sub-cellular distributions of NLRP3, ASC, and pro-Casp-1, and is co-localized with the inflammasome components to form large spots through binding to NLRP3. It has been demonstrated that distribution of NLRP3 as spots is a character of the formation of inflammasome complex [6]. Thus, our results suggested that 3D may regulate the assembly of NLRP3 inflammasome.

In the absence of stimulation, NLRP3 is homo-oligomerized, which undergo conformational changes to form active inflammasome complex by interacting ASC upon stimulation [31]. 3D up-regulates the association of NLRP3 with ASC, suggesting that it is involved in the formation of inflammasome complex. The oligomerization of ASC is critical for Casp-1 activation and inflammasome function [24]. In the presence of NLRP3, oligomerization of ASC is significantly stimulated by 3D, indicating that 3D may facilitate the assembly of NLRP3 inflammasome through interacting with NLRP3. More interestingly, 3D, NLRP3, and ASC together formed a ring-like structure, in which 3D interacts with NLRP3 that subsequently interacts with ASC. We observed that in the ring-like complex, 3D is located inside, followed by the 3D-NLRP3 complex and then the NLRP3-ASC complex, and finally ASC is located outside of the structure. It has been reported that the inflammasome complex is assembled by the formation of a ring-like organization [32]. Taken together, we demonstrated that 3D facilitates the assembly of NLRP3 inflammasome complex by the formation of a “3D-NLRP3-ASC” structure through direct binding to NLRP3.

In conclusion, we revealed a novel mechanism by which EV71 stimulates the activation of NLRP3 inflammasome by the virus-encoded 3D RNA polymerase. More importantly, 3D interacts directly with NLRP3 to facilitate the assembly of NLRP3 inflammasome complex by forming a “3D-NLRP3-ASC” ring-like structure. During the formation of the special structure, 3D binds to the LRR domain of NLRP3 that subsequently interacts with ASC through the PYRIN domain, ASC in turn binds to pro-Casp-1 by the CARD domain and activates Casp-1 (p20/p10), which finally stimulates the cleavage and release of IL-1β(p17). Thus, in this specific ring-like structure, the viral protein sites in the center, followed by NLRP3 in the middle and ASC then locates outside. IL-1β acts as an important mediator of inflammation by stimulating the activation of immune cells and the production of many secondary pro-inflammatory cytokines [15], which play important roles in the development of inflammation and associated diseases [51]. Thus, this study discovers a new role of 3D as an important regulator in the activation of inflammatory response, reveals a novel mechanism underlying the regulation of inflammasome assembly mediated by viral invasion, and would provide new insights into development of agent for the treatment and prevention of viral associated inflammation and diseases.

## Materials and Methods

### Blood samples

Blood samples of healthy donors were randomly collected from Wuhan blood donation center (Wuhan, China). To isolate peripheral blood mononuclear cells (PBMCs), blood cells were separated from blood samples and diluted in RPMI-1640 purchased from Gibco (Grand Island, NY, USA). Diluted blood cells (5 ml) were added gently to a 15 ml centrifuge tube with 5 ml lymphocyte separation medium (#50494) purchased from MP Biomedicals (California, USA), and centrifuged at 2,000×*g* for 10 min at room temperature (RT). The middle layer was transferred to another new centrifuge tube and diluted with RPMI-1640. The remaining red blood cells were removed using red blood cell lyses buffer purchased from Sigma-Aldrich (St. Louis, MO, USA). The pure PBMCs were centrifuged at 1,500×*g* for 10 min at RT and cultured in RPMI-1640.

The study was conducted according to the principles of the Declaration of Helsinki and approved by the Institutional Review Board of the College of Life Sciences, Wuhan University in accordance with its guidelines for the protection of human subjects. The Institutional Review Board of the College of Life Sciences, Wuhan University, approved the collection of blood samples for this research, in accordance with guidelines for the protection of human subjects. Written informed consent was obtained from each participant.

### Cell lines and cultures

Human rhabdomyosacroma cell line (RD) and human embryonic kidney cell line (HEK 293T) were purchased from American Type Culture Collection (ATCC) (Manassas, VA, USA). Human monocytic cells (THP-1) was a gift from Dr. Bing Sun of Institute of Biochemistry and Cell Biology, Shanghai Institute for Biological Sciences.

THP-1 cells were cultured in RPMI 1640 medium supplemented with 10% heat-inactivated fetal bovine serum (FBS), 100 U/ml penicillin, and 100 μg/ml streptomycin sulfate. RD and HEK293T cell lines were cultured in Dulbecco modified Eagle medium (DMEM) purchased from Gibco (Grand Island, NY, USA) supplemented with 10% fetal bovine serum (FBS), 100 U/ml penicillin, and 100 μg/ml streptomycin sulfate. Cells were maintained in an incubator at 37°C in a humidified atmosphere of 5% CO_2_.

### Reagents

Lipopolysaccharide (LPS), ATP, phorbol-12-myristate-13-acetate (TPA), and dansylsarcosine piperidinium salt (DSS) were purchased from Sigma-Aldrich (St. Louis, MO, USA). RPMI 1640 and Dulbecco modified Eagle medium (DMEM) were obtained from Gibco (Grand Island, NY, USA). Nigericin was obtained from InvivoGene Biotech Co., Ltd. (San Diego, CA, USA). Antibody against Flag (F3165) and monoclonal mouse anti-GAPDH (G9295) were purchased from Sigma. Monoclonal rabbit anti-NLRP3 (D2P5E), monoclonal rabbit anti-IL-1β (D3U3E), monoclonal rabbit anti-caspase-1 (catalog no. 2225) and monoclonal rabbit anti-eIF4G (C45A4) were purchased from Cell Signaling Technology (Beverly, MA, USA). Monoclonal mouse anti-ASC (sc-271054) and polyclonal rabbit anti-IL-1β (sc-7884) were purchased from Santa Cruz Biotechnology (Santa Cruz, CA, USA). Monoclonal mouse anti-NLRP3 (ALX-804-818) was purchased from Enzo Life Sciences (Shang Hai, China) to detection endogenous NLRP3 in THP1 cells by immunofluorescence microscopy. Polyclonal rabbit anti-3D antibody was produced by ABclonal Technology (Wuhan, China). Lipofectamine 2000, normal rabbit IgG and normal mouse IgG were purchased from Invitrogen Corporation (CA, USA).

### Viruses

In this study we used the Xiangyang strain of EV71 (GenBank accession number JN230523.1), which was previously isolated by our group [52,53]. The virus was adsorbed at 37°C for 2 h and the unbound virus was washed away. Infected cells were cultured in fresh medium supplemented with 2% FBS. For the preparation of UV-inactivated EV71, the virus was dispersed in a tissue culture dish, and a compact UV lamp was placed directly above the dish for 30 min. For the preparation of heat-inactivated EV71, the virus was incubated at 65°C for 30 min to completely inhibit the activity of EV71. Virus titration was performed using RD cells in 96-well plates and expressed as the 50% tissue culture infectious dose (TCID_50_) per unit volume. Sendai virus (SeV) strain was a gift from Dr. Hongbing Shu of Wuhan University. HCV genotype 2a strain JFH-1 was kindly provided by Takaji Wakita.

### Isolation of EV71 virus RNA

RD cells were infected with EV71 virus at an infection of 0.1 PFU/cell. The supernatant was collected when cells showed maximal cytopathic effect from viral infection, centrifuged at 2,500 rpm for 30min, and then passed through 0.4μm filters. We used the E.Z.N.Z. viral RNA Kit for the isolation of EV71 virus RNA from the cell culture supernatant. The concentration of viral RNA was measured by NanoDrop 2000 which was purchased from Thermo scientific.

### Stimulation of THP-1 macrophages

THP-1 cells were differentiated to macrophages with 60 nM phorbol-12-myristate-13-acetate (TPA) for 12–14 h, and cells were cultured for 24 h without TPA. The differentiated cells were then stimulated in 6 cm plates with EV71 virus, Lipopolysaccharide (LPS), Nigericin, or ATP. Supernatants were collected for measurement of IL-1β by ELISA. Cells were harvested for real-time PCR or immunoblot analysis.

### Plasmid construction

The cDNAs encoding human NLRP3, ASC, pro-Casp-1, and IL-1β were obtained by reverse transcription of total RNA from TPA-differentiated THP-1 cells, followed by PCR using specific primers. The cDNAs were sub-cloned into pcDNA3.1(+) and pcDNA3.1(+)-3×Flag vector. The pcDNA3.1(+)-3×Flag vector was constructed from pcDNA3.1(+) vector through inserting the 3×Flag sequence between the *Nhe*I and *Hind*III site. The primers used in this study are shown in S1 Table.

To construct plasmids expressing EV71 proteins 2A, 2B, 3C, 3A, 3C, and 3D, corresponding fragments of EV71 cDNA were cloned into pEGFPC1 between the *Hind*III and *Sal*I sites, resulting in green fluorescent protein (GFP) fusion protein. To construct pCAggs-HA-3D, the EV71 3D region was sub-cloned into pCAggs-HA vector using the *EcoR*I and *Kpn*I sites. To construct pGEX6p-1-3D, the EV71 3D region was sub-cloned into pGEX6p-1 vector using *BamH*I and *EcoR*I sites. The PYRIN, NACHT, and LRR domain of NLRP3 protein was cloned into pcDNA3.1(+)-3×Flag vector using specific primers shown in S1 Table.

### Lentivirus production and infection

The targeting sequences of shRNAs for the human NLRP3, ASC, and caspase-1 were as follows: sh-NLRP3: 5’-CAGGTTTGACTATCTGTTCT-3’; sh-ASC: 5’-GATGCGGAAGCTCTTCAGTTTCA-3’; sh-caspase-1: 5’-GTGAAGAGATCCTTCTGTA-3’. A PLKO.1 vector encoding shRNA for a negative control (Sigma-Aldrich, St. Louis, MO, USA) or a specific target molecule (Sigma-Aldrich) was transfected into HEK293T cells together with psPAX2 and pMD2.G with Lipofectamine 2000. We using the 3*Flag sequence to replace the GFP protein in the pLenti CMV GFP Puro vector (Addgene, 658–5) for adding some Restriction Enzyme cutting site (*Xba*I-*Eco*RV-*BstB*I-*Bam*HI) before the 3×Flag tag. Then the pLenti vector encoding EV71 3D protein was transfected into HEK293T cells together with psPAX2 and pMD2.G with Lipofectamine 2000. The primers were shown in S1 Table. Culture supernatants were harvested 36 h and 60 h after transfection and then centrifuged at 2,200rpm for 15 min. THP-1 cells were infected with the supernatants contain lentiviral particles in the presence of 4 μg/ml polybrene (Sigma). After 48 h of culture, cells were selected by 1.5 μg/ml puromycin (Sigma). The results of each sh-RNA-targeted protein and the lenti-3D protein were detected by real-time PCR and/or immunoblot analysis.

### Enzyme-Linked Immunosorbent Assay (ELISA)

The concentrations of IL-1β in culture supernatants were measured by ELISA kit (BD Biosciences, San Jose, CA).

### Measurement of activated caspase-1 and mature IL-1β

The supernatant of the cultured cells was collected for 1 ml in the cryogenic vials (Corning). The supernatant was frozen in -80°C for 4 h. The Rotational Vacuum concentrator machine that was purchase from Martin Christ was used for the freeze drying. The drying product was dissolved in 100 μl PBS and mixed with SDS loading buffer for western blotting analysis with antibodies for detection of activated caspase-1 (D5782 1:500; Cell Signaling) or mature IL-1β (Asp116 1:500; Cell Signaling). Adherent cells in each well were lysed with the lysis buffer described below, followed by immunoblot analysis to determine the cellular content of various protein.

### Western blot analysis

The HEK293T whole-cell lysates were prepared by lysing cells with buffer (50 mM Tris-HCl, pH7.5, 300 mM Nacl, 1% Triton-X, 5 mM EDTA, and 10% glycerol). The TPA-differentiated THP-1 cells lysates were prepared by lysing cells with buffer (50 mM Tris-HCl, pH7.5, 150 mM Nacl, 0.1% Nonidetp40, 5 mM EDTA, and 10% glycerol). Protein concentration was determined by Bradford assay (Bio-Rad, Hercules, CA, USA). Cultured cell lysates (30 μg) were electrophoresed in an 8–12% SDS-PAGE gel and transferred to a PVDF membrane (Millipore, MA, US). PVDF membranes were blocked with 5% skim milk in phosphate buffered saline with 0.1% Tween 20 (PBST) before being incubated with the antibody. Protein band were detected using a Luminescent image Analyzer (Fujifilm LAS-4000).

### Co-immunoprecipitation assays

The HEK293T whole-cell lysates were prepared by lysing cells with buffer (50 mM Tris-HCl, pH7.5, 300 mM Nacl, 1% Triton-X, 5 mM EDTA, and 10% glycerol). The TPA-differentiated THP-1 cells lysates were prepared by lysing cells with buffer (50 mM Tris-HCl, pH7.5, 150 mM Nacl, 0.1% Nonidetp40, 5 mM EDTA, and 10% glycerol). Lysates were immunoprecipitated with control mouse immunoglobulin G (IgG) (Invitrogen) or anti-Flag antibody (Sigma, F3165) with Protein-G Sepharose (GE Healthcare, Milwaukee, WI, USA).

### Confocal microscopy

TPA-differentiated THP-1 cells was cultured or infected by the EV71 (MOI = 20) virus for 24 h. Cells were fixed in 4% paraformaldehyde at room temperature for 15 min. After being washed three times with PBS, permeabilized with PBS containing 0.1% Triton X-100 for 5 min, washed three times with PBS, and finally blocked with PBS containing 5% BSA for 1 h. The cells were then incubated with the monoclonal mouse IgG1 anti-NLRP3 antibody (ALX-804-818-C100; Enzo life Sciences) and the polyclonal rabbit anti-3D antibody (ABclonal technology) overnight at 4°C, followed by incubation with FITC-conjugate donkey anti-mouse IgG and Dylight 649-conjugate donkey anti-rabbit IgG (Abbkine) for 1 h. After washing three times, cells were incubated with DAPI solution for 5 min, and then washed three more times with PBS. Finally, the cells were analyzed using a confocal laser scanning microscope (Fluo View FV1000; Olympus, Tokyo, Japan).

### Real-time PCR

Total RNA was extracted with TRIzol reagent (Invitrogen), following the manufacturer’s instructions. Real-time quantitative RT-PCR was performed using the Roche LC480 and SYBR RT-PCR kits (DBI Bio-science, Ludwigshafen, Germany) in a reaction mixture of 20 μl SYBR Green PCR master mix, 1 μl DNA diluted template, and RNase-free water to complete the 20 μl volume. Real-time PCR primers were designed by Primer Premier 5.0 and their sequences were was as follows: VP1 forward, 5’-CCCTTTAGTGGTTAGGATTT-3’, VP1 reverse, 5’-CACCAGTTGGTTTAATGGAG-3’; NLRP3 forward, 5’-AAGGGCCATGGACTATTTCC-3’, NLRP3 reverse, 5’-GACTCCACCCGATGACAGTT-3’; ASC forward, 5’-AACCCAAGCAAGATGCGGAAG-3’, ASC reverse, 5’-TTAGGGCCTGGAGGAGCAAG-3’; caspase-1 forward, 5’-TCCAATAATGCAAGTCAAGCC-3’, caspase-1 reverse, 5’-GCTGTACCCCAGATTTTGTAGCA-3’; IL-1β forward, 5’-CACGATGCACCTGTACGATCA-3’, IL-1β reverse, 5’-AGCACTGAAAGCATGA-3’, TNF-α forward, 5’-GGGTTTGCTACAACATGG-3’, TNF-α reverse, 5’-AAGAACTTAGATGTCAGTGC-3’, IL-8 forward, 5’-ACTTCTCCACAACCCTCTGC-3’, IL-8 reverse, 5’-GTTGCTCCATATCCTGTCCCT-3’; GAPDH forward, 5’-AAGGCTGTGGGCAAGG-3’, GAPDH reverse, 5’-TGGAGGAGTGGGTGTCG-3’.

### GST pull down assays

The construct pGEX6p-1-3D plasmid and pGEX6p-1-LRR were transfected into *Escherichia coli* strain BL21. After growing in LB medium at 3°C until the OD600 reached 0.6–0.8, Isopropyl β-D-1-thiogalactopyranoside (IPTG) was added to a final concentration of 0.1 mM and the cultures grew for an additional 16 h at 16°C for GST-3D protein. Isopropyl β-D-1-thiogalactopyranoside (IPTG) was added to a final concentration of 1 mM and the cultures grew for an additional 4 h at 37°C for GST-LRR protein. And then the GST protein, GST-3D protein and GST-LRR protein were purified from the E. coli bacteria. For GST-3D pull-down assay, glutathione-Sepharose beads (Novagen) were incubated with GST-3D or GST protein. After washed with phosphate-buffered saline (PBS), these beads were incubated with cell lysates from HEK293T which were transfected with plasmids encoding Flag-NLRP3, Flag-ASC and Flag-pro-caspase-1 for 4 h at 4°C. The precipitates were washed three times, boiled in 2×SDS loading buffer, separated by 10% SDS-PAGE, immunoblotted with anti-GST, anti-Flag, and ant-ASC antibody. It was the same for the GST-LRR pull down assay.

### Yeast two-hybrid analysis

Saccharomyces cerevisiae strain AH109, control vectors pGADT7, pGBKT7, pGADT7-T, pGBKT7-lam, and pGBKT7-p53 were purchased from Clontech (Mountain View, CA, USA). Yeast strain AH109 was co-transformed with the combination of the pGADT7 and the pGBKT7 plasmids. Transformed yeast cells containing both plasmids were first grown on SD-minus Trp/Leu plates (DDO) to maintain the two plasmids and then were sub-cloned replica plated on SD-minus Trp/Leu/Ade/His plate (QDO).

### ASC oligomerization detection

The TPA-differentiated THP-1 cells were lysed by buffer (50 mM Tris, pH7.5, 150 mM Nacl, 1% Nonidetp40, 5 mM EDTA, and 10% glycerol) at 4°C. The transfected HEK293T cells were lysed by buffer (50 mM Tris-HCl, pH7.5, 300 mM Nacl, 1% Triton-X, 5 mM EDTA, and 10% glycerol). Lysates were centrifugated at 6000rpm for 15 min. The supernatants of the lysates were mixed with SDS loading buffer for western blot analysis with antibody against ASC. The pellets of the lysates were washed with PBS for three times and cross-linked using fresh DSS (2 mM, sigma) at 37°C for 30 min. The cross-linked pellets were then spanned down and mixed with SDS loading buffer for western blotting analysis.

### Statistical analyses

All experiments were reproducible and repeated at least three times with similar results. Parallel samples were analyzed for normal distribution using Kolmogorov-Smirnov tests. Abnormal values were eliminated using a follow-up Grubbs test. Levene’s test for equality of variances was performed, which provided information for Student’s *t*-tests to distinguish the equality of means. Means were illustrated using histograms with error bars representing the SD; a P value of <0.05 was considered statistically significant.

## Supporting Information

S1 Fig Determination of the expression of EV71 VP1 mRNA during viral infection in THP-1 macrophages. (**A**) TPA-differentiated THP-1 macrophages were stimulated by LPS (1 μg/ml) for 6 h plus 2 μM Nigericin for 30 min, and then infected with EV71 at MOI = 10 for 24 h or 48 h. The mRNA levels for EV71 VP1were quantified by real-time PCR. (**B**) TPA-differentiated THP-1 macrophages were stimulated by LPS (1 μg/ml) for 6 h plus 2 μM Nigericin for 30 min, and then infected with EV71 for 24 h at MOI = 5, 10, or 20. The mRNA levels for EV71 VP1were quantified by real-time PCR. (**C**) TPA-differentiated THP-1 cells were treated with LPS (1 μg/ml) for 6 h or infection with EV71. The mRNA levels for IL-1β, IL8, TNF-α, and EV71 VP1 were quantified by qRT-PCR. (**D**) ASC oligomerization in TPA-differentiated THP-1 cells which were infected by EV71. TPA-differentiated THP-1 cells were treated with the 2 μM Nigericin for 2 h as a positive control.
(TIF)

S2 Fig Analyses of the efficiency of short hairpin RNAs (shRNA) in stable THP-1 cell lines. (**A** to **D**) TPA-differentiated THP-1 macrophages were targeted with negative control shRNA (sh-NC) or shRNA specific to NLRP3 (sh-NLRP3), ASC (sh-ASC), or pro-caspase-1 (sh-Casp1), respectively. The levels of NLRP3 (A), ASC (B), and pro-Casp-1 (C) mRNAs in the stable cell lines were determined by qRT-PCR. The levels of NLRP3, ASC, and pro-Casp-1 protein expression in the stable cell lines were determined by Western blots (D).
(TIF)

S3 Fig Determination of the expression of EV71 VP1 mRNA during viral infection in human PBMCs. (**A**) Human PBMCs were treated with LPS at 1 μg/ml for 6 h, infected with EV71 at an MOI of 5 for 36 h, or inoculated with UV-inactivated (UV-inact.) or heat-inactivated (heat-inact.) EV71 at an MOI of 5 for 36 h. The mRNA levels for EV71 VP1 were quantified by qRT-PCR. (**B**) TPA-differentiated THP-1 cells were stimulated for 6 h with Lipo (Control), EV71 RNA (5 μg/ml) plus Lipo, HCV RNA (5 μg/ml) plus Lipo or 5 μg/ml poly dA:dT plus Lipo (positive control). The mRNA levels for IL-1β, TNF-α, EV71 VP1, and HCV NS5B were quantified by qRT-PCR.
(TIF)

S4 Fig Determination of the interaction of EV71 3D protein with NLRP3 LRR, PYRIN, and NACHT domains. (**A**) Identification of NLRP3 inflammasomes three components and NLRP3 protein three domains-EV71 3D protein interaction by yeast two-hybrid analysis. Yeast strain AH109 cells were transformed with the combination of BD and AD plasmid, as indicated. Transformed yeast cells were first grown on the SD-minus Trp/Leu plates for three days. The colony of yeast was then streaked on SD-minus Trp/Leu/Ade/His plates (QDO). BD-p53 and AD-T was used as a positive control and BD-lam and AD-T as a negative control. (**B**) Identification of NLRP3 LRR domain-EV71 3D protein interaction by yeast two-hybrid analysis. (**C**) Diagrams of the structures of NLRP3 protein, NLRP3 PYRIN domain, NLRP3 NACHT domain, and NLRP3 LRR domain. The numbers indicated the locations of aa sequences.
(TIF)

S5 Fig Determination of the interaction of 3D protein NLRP3 and ASC proteins. HEK293T cells were co-transfected with plasmid expressing HA-3D and plasmids encoding Flag-NLRP3 or Flag-ASC. Lysates were subjected to IP using IgG or anti-Flag antibody (top), and then analyzed by Western blot using anti-3D antibody and anti-Flag antibody. Lysates were also analyzed directly (30 μg protein, bottom) by Western blot using anti-3D antibody and anti-Flag antibody (as input).
(TIF)

S6 Fig EV71 infection alters subcellular distribution of ASC. TPA-differentiated THP-1 macrophages were infected with or without EV71. The distributions of ASC (green) and nucleus marker DAPI (blue) were analyzed with confocal microscopy.
(TIF)

S1 Table Primers used in this study to construct the plasmids. (DOC)
