# Correction: EV71 3D Protein Binds with NLRP3 and Enhances the Assembly of Inflammasome Complex

**DOI:** 10.1371/journal.ppat.1006921

**Published:** 2018-03-12

**Authors:** Wenbiao Wang, Feng Xiao, Pin Wan, Pan Pan, Yecheng Zhang, Fang Liu, Kailang Wu, Yingle Liu, Jianguo Wu

The following information is missing from the Funding section: This work was supported by grants from National Natural Science Foundation of China (81730061, 31230005, 81471942, 31200134, and 31270206), and the National Mega Project on Major Infectious Disease Prevention (2017ZX10103005). The funders had no role in study design, data collection and analysis, decision to publish, or preparation of the manuscript.
