# Supplementary figures and images for: EV71 3D Protein Binds with NLRP3 and Enhances the Assembly of Inflammasome Complex
Source: PLoS Pathog. 2017 Jan 6;13(1):e1006123. doi: 10.1371/journal.ppat.1006123 (PMC5245909; doi:10.1371/journal.ppat.1006123)

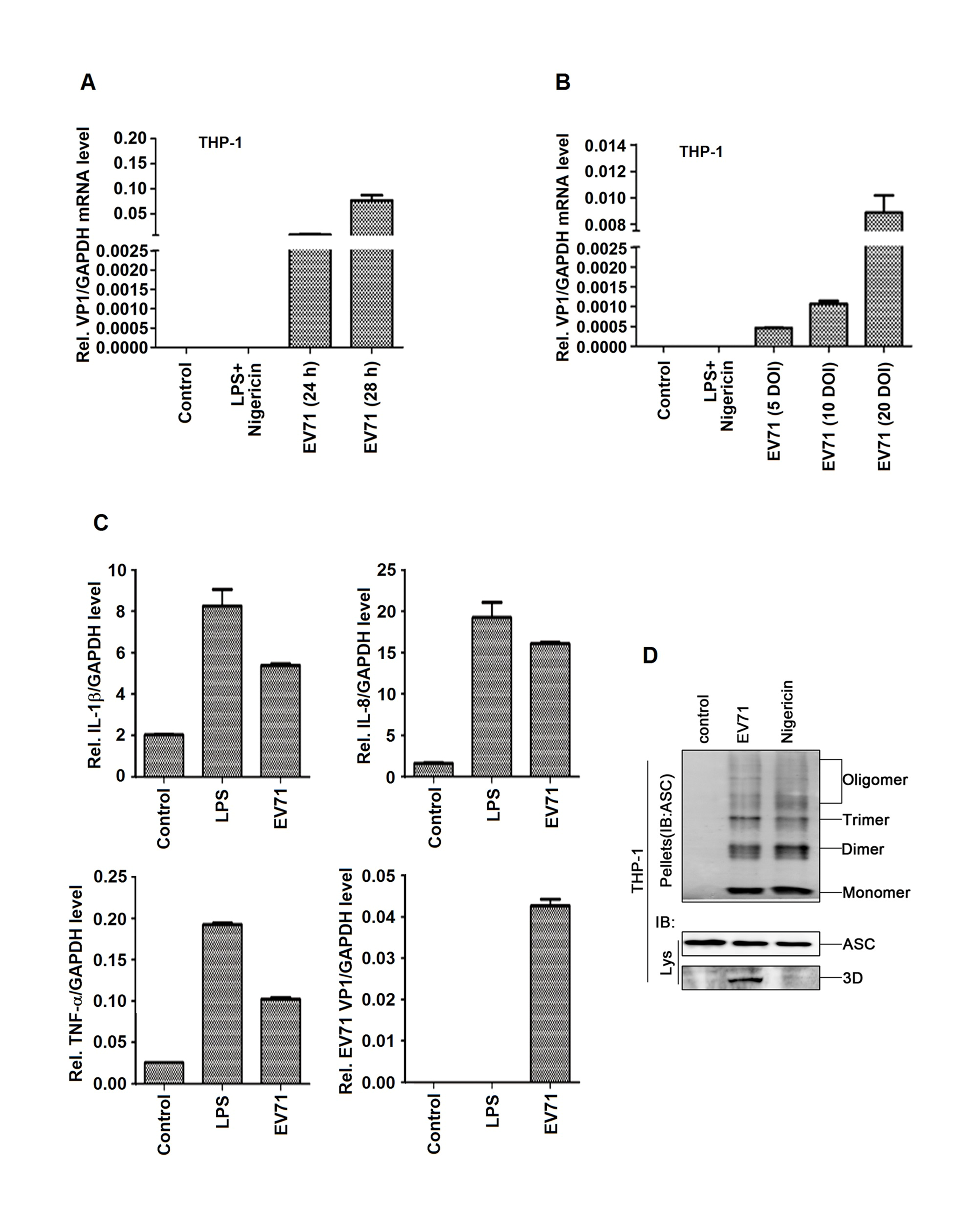

Supplement: S1 Fig — (A) TPA-differentiated THP-1 macrophages were stimulated by LPS (1 μg/ml) for 6 h plus 2 μM Nigericin for 30 min, and then infected with EV71 at MOI = 10 for 24 h or 48 h. The mRNA levels for EV71 VP1were quantified by real-time PCR. (B) TPA-differentiated THP-1 macrophages were stimulated by LPS (1 μg/ml) for 6 h plus 2 μM Nigericin for 30 min, and then infected with EV71 for 24 h at MOI = 5, 10, or 20. The mRNA levels for EV71 VP1were quantified by real-time PCR. (C) TPA-differentiated THP-1 cells were treated with LPS (1 μg/ml) for 6 h or infection with EV71. The mRNA levels for IL-1β, IL8, TNF-α, and EV71 VP1 were quantified by qRT-PCR. (D) ASC oligomerization in TPA-differentiated THP-1 cells which were infected by EV71. TPA-differentiated THP-1 cells were treated with the 2 μM Nigericin for 2 h as a positive control. (TIF) [file ppat.1006123.s001.tif]

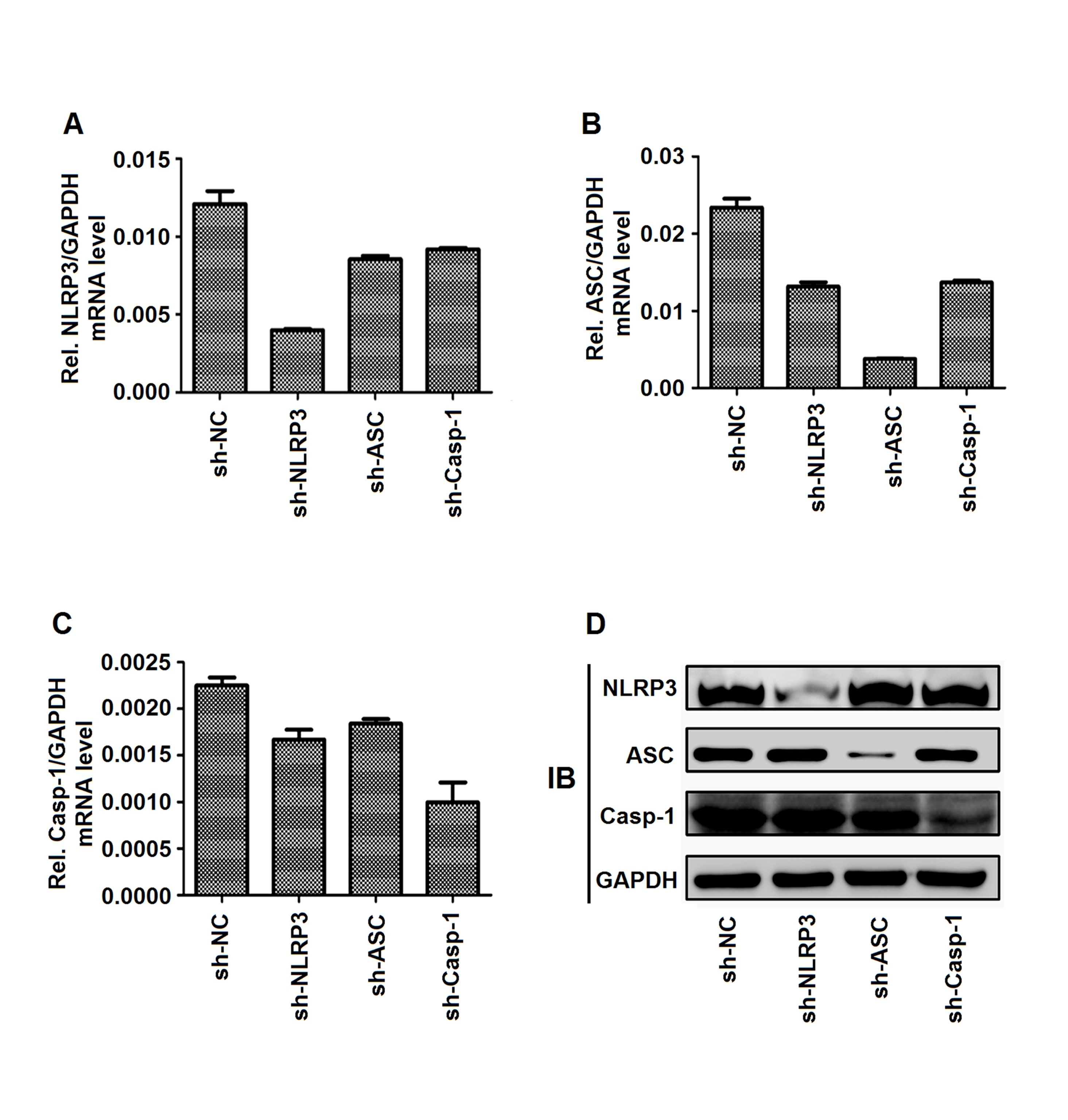

Supplement: S2 Fig — (A to D) TPA-differentiated THP-1 macrophages were targeted with negative control shRNA (sh-NC) or shRNA specific to NLRP3 (sh-NLRP3), ASC (sh-ASC), or pro-caspase-1 (sh-Casp1), respectively. The levels of NLRP3 (A), ASC (B), and pro-Casp-1 (C) mRNAs in the stable cell lines were determined by qRT-PCR. The levels of NLRP3, ASC, and pro-Casp-1 protein expression in the stable cell lines were determined by Western blots (D). (TIF) [file ppat.1006123.s002.tif]

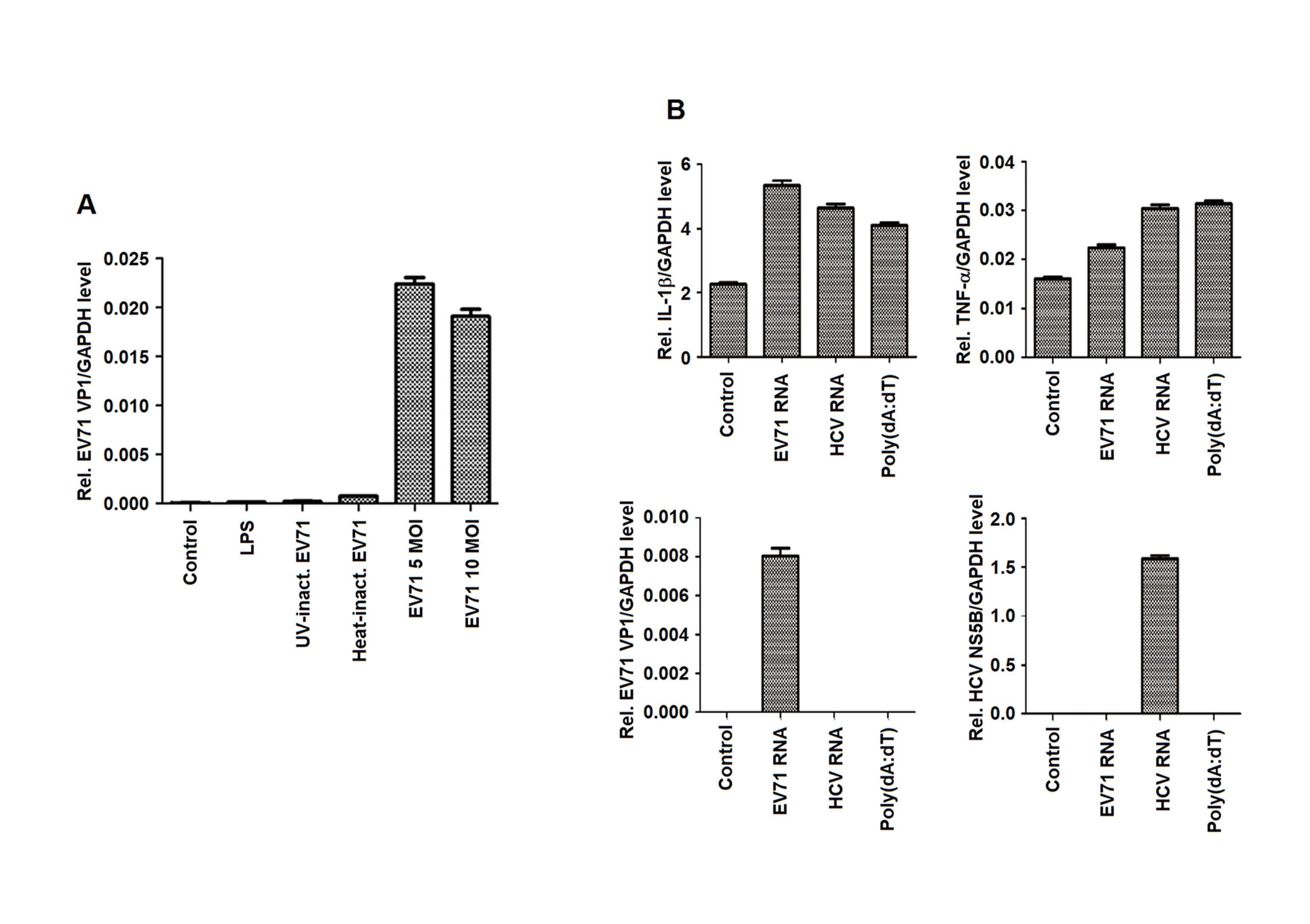

Supplement: S3 Fig — (A) Human PBMCs were treated with LPS at 1 μg/ml for 6 h, infected with EV71 at an MOI of 5 for 36 h, or inoculated with UV-inactivated (UV-inact.) or heat-inactivated (heat-inact.) EV71 at an MOI of 5 for 36 h. The mRNA levels for EV71 VP1 were quantified by qRT-PCR. (B) TPA-differentiated THP-1 cells were stimulated for 6 h with Lipo (Control), EV71 RNA (5 μg/ml) plus Lipo, HCV RNA (5 μg/ml) plus Lipo or 5 μg/ml poly dA:dT plus Lipo (positive control). The mRNA levels for IL-1β, TNF-α, EV71 VP1, and HCV NS5B were quantified by qRT-PCR. (TIF) [file ppat.1006123.s003.tif]

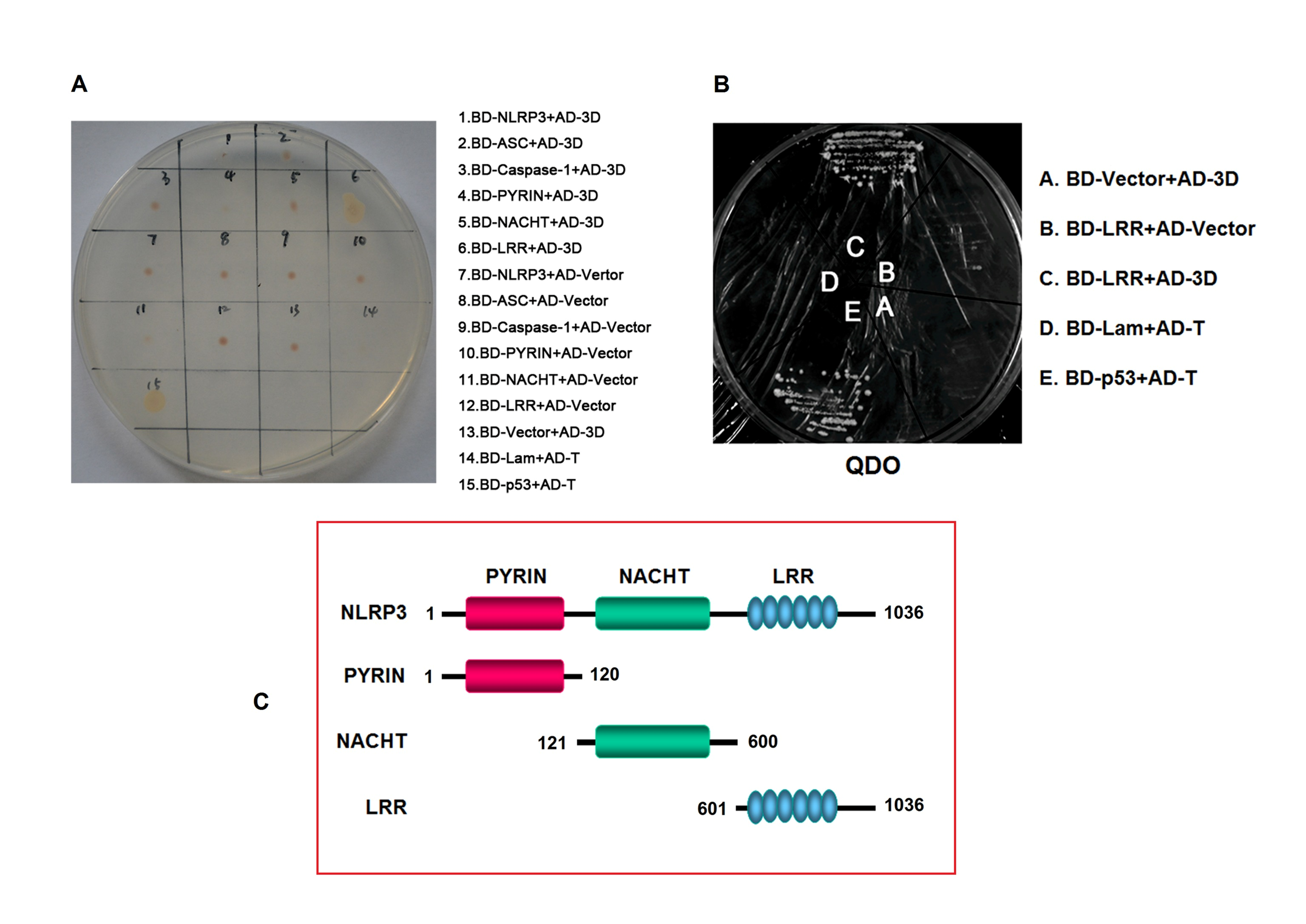

Supplement: S4 Fig — (A) Identification of NLRP3 inflammasomes three components and NLRP3 protein three domains-EV71 3D protein interaction by yeast two-hybrid analysis. Yeast strain AH109 cells were transformed with the combination of BD and AD plasmid, as indicated. Transformed yeast cells were first grown on the SD-minus Trp/Leu plates for three days. The colony of yeast was then streaked on SD-minus Trp/Leu/Ade/His plates (QDO). BD-p53 and AD-T was used as a positive control and BD-lam and AD-T as a negative control. (B) Identification of NLRP3 LRR domain-EV71 3D protein interaction by yeast two-hybrid analysis. (C) Diagrams of the structures of NLRP3 protein, NLRP3 PYRIN domain, NLRP3 NACHT domain, and NLRP3 LRR domain. The numbers indicated the locations of aa sequences. (TIF) [file ppat.1006123.s004.tif]

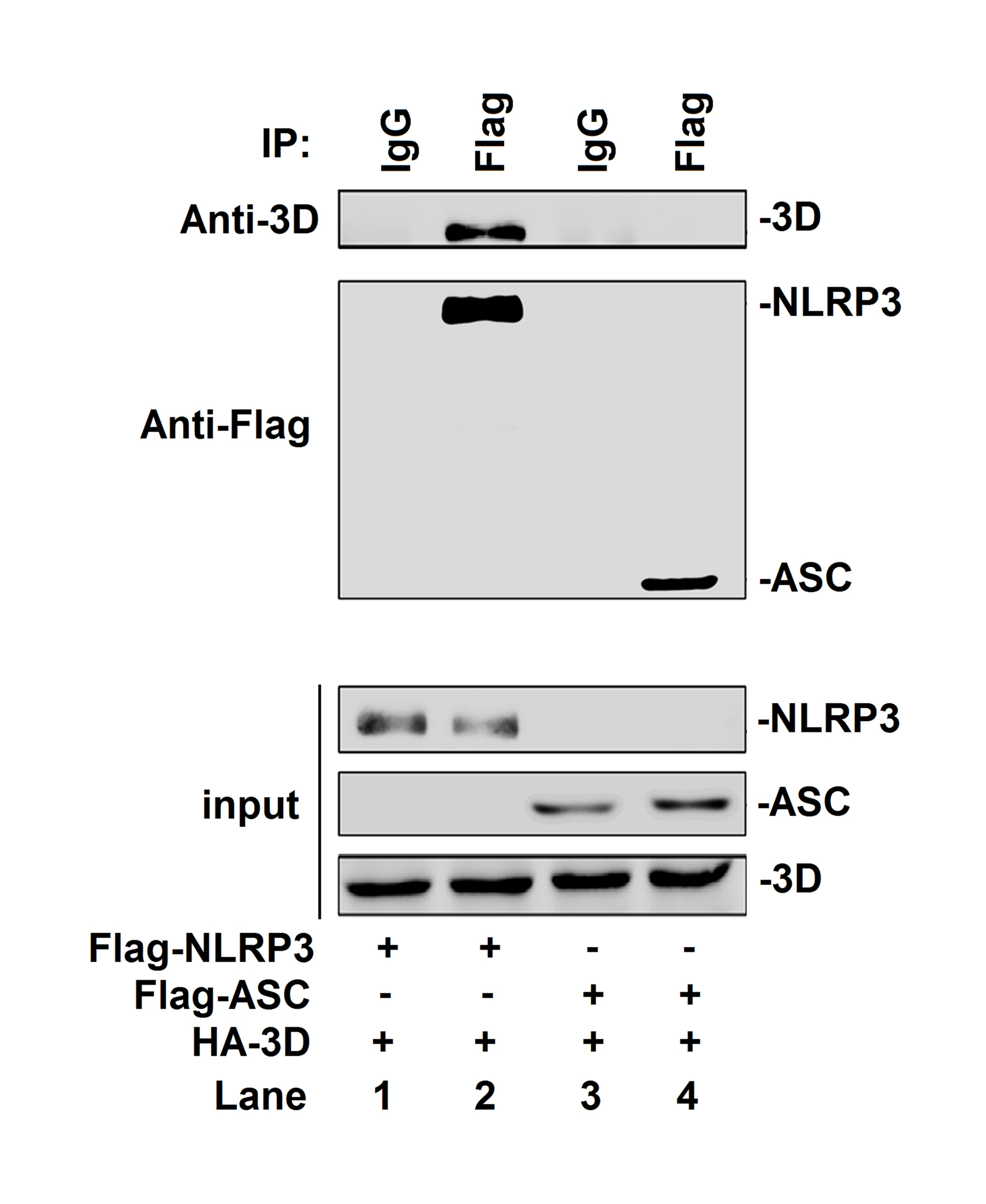

Supplement: S5 Fig — HEK293T cells were co-transfected with plasmid expressing HA-3D and plasmids encoding Flag-NLRP3 or Flag-ASC. Lysates were subjected to IP using IgG or anti-Flag antibody (top), and then analyzed by Western blot using anti-3D antibody and anti-Flag antibody. Lysates were also analyzed directly (30 μg protein, bottom) by Western blot using anti-3D antibody and anti-Flag antibody (as input). (TIF) [file ppat.1006123.s005.tif]

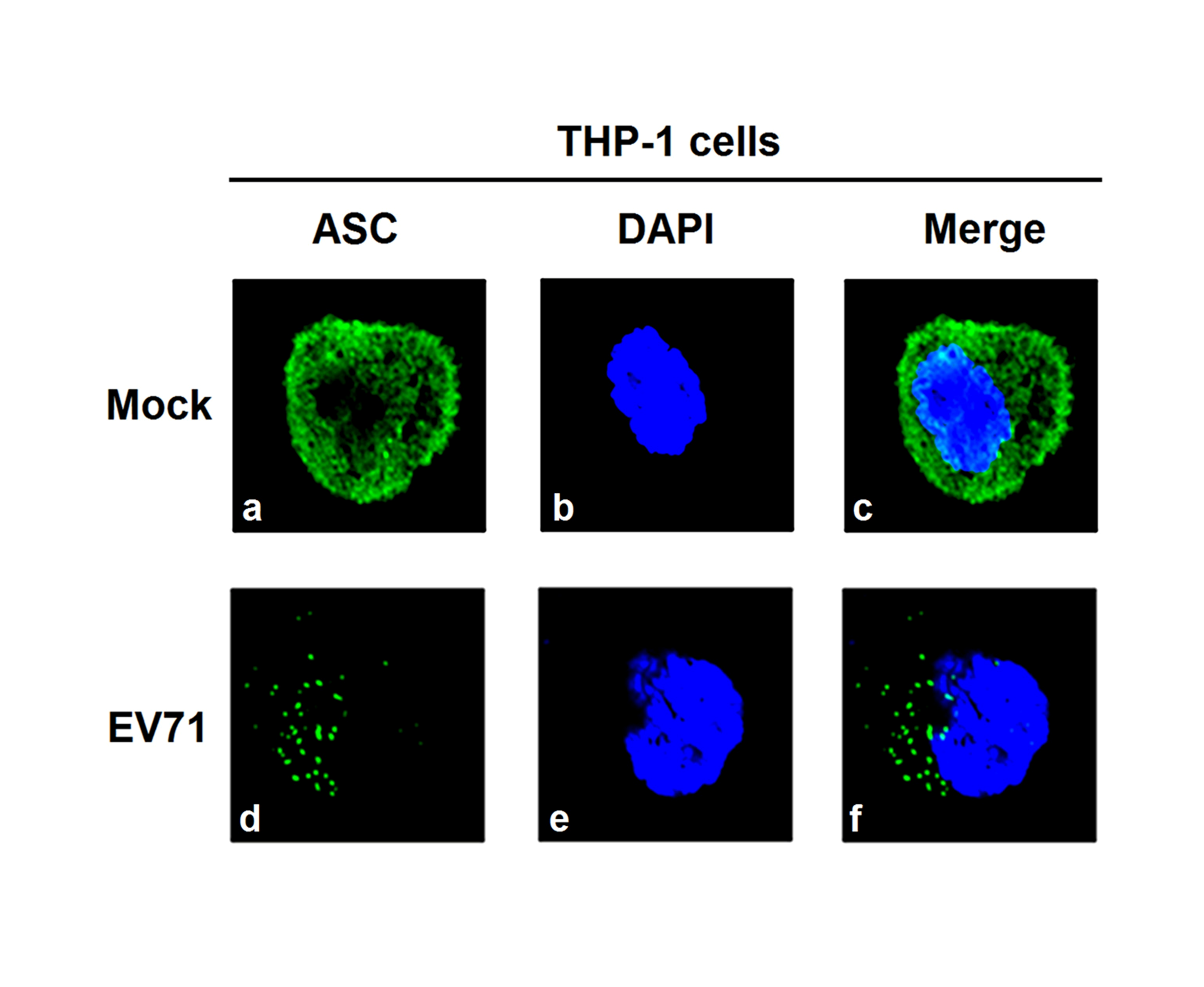

Supplement: S6 Fig — TPA-differentiated THP-1 macrophages were infected with or without EV71. The distributions of ASC (green) and nucleus marker DAPI (blue) were analyzed with confocal microscopy. (TIF) [file ppat.1006123.s006.tif]
