# Supplementary material for: EV71 3D Protein Binds with NLRP3 and Enhances the Assembly of Inflammasome Complex
Source: PLoS Pathog. 2017 Jan 6;13(1):e1006123. doi: 10.1371/journal.ppat.1006123 (PMC5245909; doi:10.1371/journal.ppat.1006123)
Supplement: S1 Table — (DOC) [file ppat.1006123.s007.doc]

**S1 Table. Primers used in this study to construct the plasmids.**

| Plasmids and gene | Sense Primer | Anti-Sense Primer |
| --- | --- | --- |
| pcDNA3.1(+)-NLRP3 | 5'-CGCGGATCCATGAAGATGGCAAGCACCCGC-3' | 5'-CCGCTCGAGCTACCAAGAAGGCTCAAAGAC-3' |
| pcDNA3.1(+)-ASC | 5'-CCGGAATTCATGGGGCGCGCGCGCGACGCCAT-3' | 5'-CCGCTCGAGTCAGCTCCGCTCCAGGTCCTCCA-3' |
| pcDNA3.1(+)-caspase1 | 5'-CGCGGATCCATGGCCGACAAGGTCCTGAAG-3' | 5'-CCGCTCGAGTTAATGTCCTGGGAAGAGGTA-3' |
| pcDNA3.1(+)-il-1BETA | 5'-CCGGAATTCATGGCAGAAGTACCTGAGCTC-3' | 5'-CCGCTCGAGTTAGGAAGACACAAATTGCAT-3' |
| pcagg-HA-3D | 5'-CCGGAATTCATGGGAGAGATCCAGTGGGTT-3' | 5'-CGGGGTACCAAATAACTCGAGCCAATTGCG-3' |
| pGEX-6p-1-3D | 5'-TTGGCGCGCATGGGAGAGATCCAGTGGGTT-3' | 5'-CCGGAATTCCTAAAATAACTCGAGCCAATT-3' |
| pGEX-6p-1-LRR | CGCGGATCCATGTCTCAGCAAATCAGGCTG | CCGCTCGAGCTACCAAGAAGGCTCAAAGAC |
| pcDNA3.1(+)-3×Flag-NLRP3 | 5'-GGCATATGATGAAGATGGCAAGCACCCG-3' | 5'-CCGGATCCCTACCAAGAAGGCTCAAAG-3' |
| pcDNA3.1(+)-3×Flag-ASC | 5'-CCGGAATTCATGGGGCGCGCGCGCGACGCCAT-3' | 5'-CCGCTCGAGTCAGCTCCGCTCCAGGTCCTCCA-3' |
| pcDNA3.1(+)-3×Flag-Caspase1 | 5'-CCGAATTCATGGCCGACAAGGTCCTGAAG-3' | 5'-CCGGATCCTTAATGTCCTGGGAAGAGG-3' |
| pcDNA3.1(+)-3×Flag-PYRIN | 5'-AAAGGATCCATGAAGATGGCAAGCACCCGC-3' | 5'-CGGCTCGAGCTATAAACCCATCCACTCCTCTTC-3' |
| pcDNA3.1(+)-3×Flag-NBD | 5'-AAAGGATCCCTGGAGTACCTTTCGAGAATCTC-3' | 5'-CCCCTCGAGCTAGATCTTGCAACTTAATTTCTTC-3' |
| pcDNA3.1(+)-3×Flag-LRR | 5'-AAAGGATCCTCTCAGCAAATCAGGCTGGAG-3' | 5'-CGGCTCGAGCTACCAAGAAGGCTCAAAGACG-3' |
| pLenti-3D | 5'-CTAGTCTAGAATGGGAGAGATCCAGTGGGTTAAGC-3' | 5'-CGCGGATCCTTAAAATAACTCGAGCCAATTGCGTC-3' |
| pGBKT7-NLRP3 | 5'-GGGAATTCCATATGATGAAGATGGCAAGCA-3' | 5'-CGCGGATCCCTACCAAGAAGGCTCAAAGAC-3' |
| pGBKT7-PYRIN | 5’-CCGGAATTCATGAAGATGGCAAGCACCCGC-3’ | 5’-CGCGGATCCCTATAAACCCATCCACTCCTC-3’ |
| pGBKT7-NACHT | 5’-CCGGAATTCATGCTGGAGTACCTTTCGAGA-3’ | 5’-CGCGGATCCCTAGATCTTGCAACTTAATTT-3’ |
| pGBKT7-LRR | 5'-CGCGGATCCATATGTCTCAGCAAATCAGGC-3' | 5'-CCGCTCGAGCTACCAAGAAGGCTCAAAGAC-3' |
| pGBKT7-ASC | 5'-CCGGAATTCATGGGGCGCGCGCGCGACGCC-3' | 5'-CGCGGATCCTCAGCTCCGCTCCAGGTCCTC-3' |
| pGBKT7-caspase-1 | 5'-CCGGAATTCATGGCCGACAAGGTCCTGAAG-3' | 5'-CGCGGATCCTTAATGTCCTGGGAAGAGGTA-3' |
| pGADT7-3D | 5'-CCGGAATTCATGGGAGAGATCCAGTGGGTT-3' | 5'-CGCGGATCCTTAAAATAACTCGAGCCAATT-3' |
